# The Nϵ‐Rule for Serine, but Not Cysteine Catalytic Triads

**DOI:** 10.1002/anie.202206945

**Published:** 2022-09-05

**Authors:** Honorata Czapinska, Matthias Bochtler

**Affiliations:** ^1^ International Institute of Molecular and Cell Biology Trojdena 4 02-109 Warsaw Poland; ^2^ Institute of Biochemistry and Biophysics of the Polish Academy of Sciences Pawinskiego 5a 02-106 Warsaw Poland

**Keywords:** Catalytic Mechanism, Esterase, Nϵ-Rule, Peptidase, Triad

## Abstract

Catalytic triads, composed of a serine or cysteine nucleophile, a histidine, and a third triad residue (typically Asp/Glu/Asn), are common in enzyme active sites and catalyze a wide variety of chemical reactions. Two types of triads can be distinguished: We refer to them as Nδ‐ or Nϵ‐configured, depending on whether the histidine imidazole Nδ or Nϵ atom is close to the nucleophile Oγ/Sγ. In this study, we have analyzed triad configuration. In structural triads, the more stable Nδ‐configuration predominates. For catalytic triads, the configuration depends on the nucleophile. When it is a cysteine residue, both configuration types occur, depending on the family. However, when the nucleophile is a serine residue, the less stable Nϵ‐configuration is almost exclusively found. We posit that the energetically less favored conformation is selected for in serine triads to facilitate the otherwise difficult proton transfer from the nucleophile to the histidine residue.

## Graphical abstract

Serine‐containing catalytic triads are almost exclusively Nϵ‐configured (the histidine Nϵ atom is next to the nucleophile). This is in stark contrast to cysteine catalytic triads (and non‐catalytic triads). It is argued that the effect stems from the p*K*_a_ difference of the histidine nitrogen atoms. The Nϵ‐triads are evolutionarily selected for at the cost of stability to help overcome the Ser‐His p*K*_a_ gradient much larger than that of the Cys‐His pair.

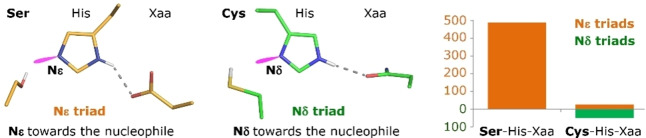

Catalytic triads are among the most common active sites in metal‐independent enzymes and an important part of nature's catalytic toolkit.[^1^, ^2^, ^3^, ^4^] Typically, they consist of a serine or cysteine nucleophile, hydrogen bonded to a histidine residue, connected via a hydrogen bond to a third triad residue, which is typically an aspartate, glutamate or asparagine (in rare cases a glutamine or histidine). It has been understood for decades that the role of the third triad residue is not only to orient the histidine imidazole, but also to make it more basic to facilitate the proton transfer from the nucleophile to the (general) base histidine.^[5]^ p*K*_a_ values in solution are higher for serine (p*K*_a_≈13.6^[6]^) and cysteine (p*K*_a_≈9.1^[7]^), than for histidine (p*K*_a_≈6.4^[7]^), suggesting that the proton transfer carries a free energy penalty. In serine catalytic triads, the penalty is large enough that the proton transfer can only take place during the reaction, as the nucleophile gets acidified by its approach to the target. For cysteine catalytic triads, the penalty is smaller and may be absent altogether in some proteins due to the local environment that can shift the p*K*_a_ value by several units.^[8]^ It has been argued that in cysteine peptidases the active site may already preexist in a Cys^−^ His^+^ ion pair form.^[9]^

Two triad configurations can be distinguished, according to the identity of the imidazole nitrogen in immediate proximity to the nucleophile. For the Nϵ‐triad, the Nϵ is near the nucleophile and is therefore an unprotonated hydrogen bond acceptor. Hence the imidazole proton must reside on the Nδ, which donates a hydrogen bond to the third triad residue. For the Nδ‐triad, the roles of Nδ and Nϵ are reversed (Figure 1).

**Figure 1:**
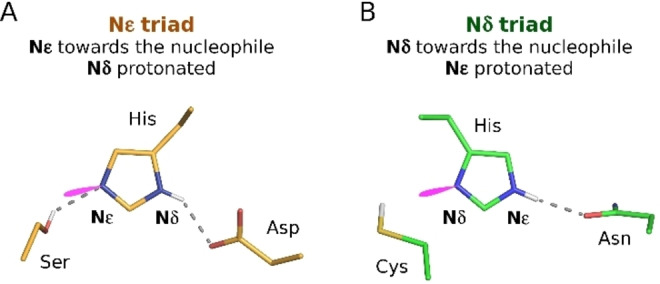
Nϵ and Nδ triad configurations. A) Trypsin (PDB 4y0y^[10]^) and B) papain (PDB 1pip^[11]^) are used as examples for two triad arrangements. Putative hydrogen bonds are indicated as dashed lines, the lone pair of the unprotonated nitrogen atom is in magenta. Varieties of orange and green are used throughout to depict Nϵ‐ and Nδ‐triads, respectively.

Nδ‐ and Nϵ‐triad configurations are widely regarded as equivalent, but ^13^C NMR studies with methylated histidine analogues show that the histidine Nϵ atom is approximately 0.4 p*K*_a_ units more basic than its Nδ atom.^[12]^ According to the Boltzmann rule, the histidine tautomer with proton on the Nϵ is therefore *kT* ln(10^Δp*K*a^) or about 0.5–0.6 kcal mol^−1^ more stable than the tautomer with proton on the Nδ (Boltzmann energy, *kT*≈0.6 kcal mol^−1^ at room temperature). This free energy difference translates into an expected 10^Δp*K*a^ or 2.5‐fold preference for the Nδ‐configuration in the absence of evolutionary selection.

Triads occur not only as a catalytic motif, but also as structural elements. Selective pressure related to catalysis should not affect such triads. Hence, their frequencies in the Protein Data Bank (PDB)^[13]^ can be used to test stability predictions. We first defined an optimal triad geometry. We assumed that a hydrogen bond donor/acceptor should be located on the line of the histidine Nδ/Nϵ sp^2^ lone pair orbital at a distance of ≈2.8 Å. We used the histidines as an anchor and scanned the PDB for triads with Oγ/Sγ and Oδ/Oϵ atoms within a 3 Å sphere around the ideal positions (Figure S1). We eliminated peptidase and esterase entries from the set (Suppl. Meth.).

To compensate for the overrepresentation of some proteins in the PDB, we carried out a weighted analysis. We clustered amino acid sequences of the triad containing protein chains using CD‐HIT^[14]^ with a 70 % sequence identity cutoff. We counted every triad case with a weight of 1/(number of instances in its cluster).

The weighted analysis indicated a 1.8, 1.9 and 1.3 fold preference for the Nδ‐triads with a serine, threonine or cysteine as the first triad residue, respectively. The ≈1.8 fold preference in the pooled triad set was in reasonable agreement with the expected ≈2.5 fold preference deduced from basicity differences between the Nδ and Nϵ atoms (Table S1).

Catalytic triads are very well characterized in peptidases. MEROPS,^[15]^ a highly curated peptidase database, classifies them based on evolutionary criteria into clans, further subdivided into families. Since some catalytic triads were structurally distorted e.g., due to the presence of inhibitors, we performed the triad search with a more generous 4 Å radius (Figure S2) and manually curated the obtained cases of false positives. Unweighted and weighted analysis was carried out separately for MEROPS families and clans (Figure 2, S3–S4).

**Figure 2:**
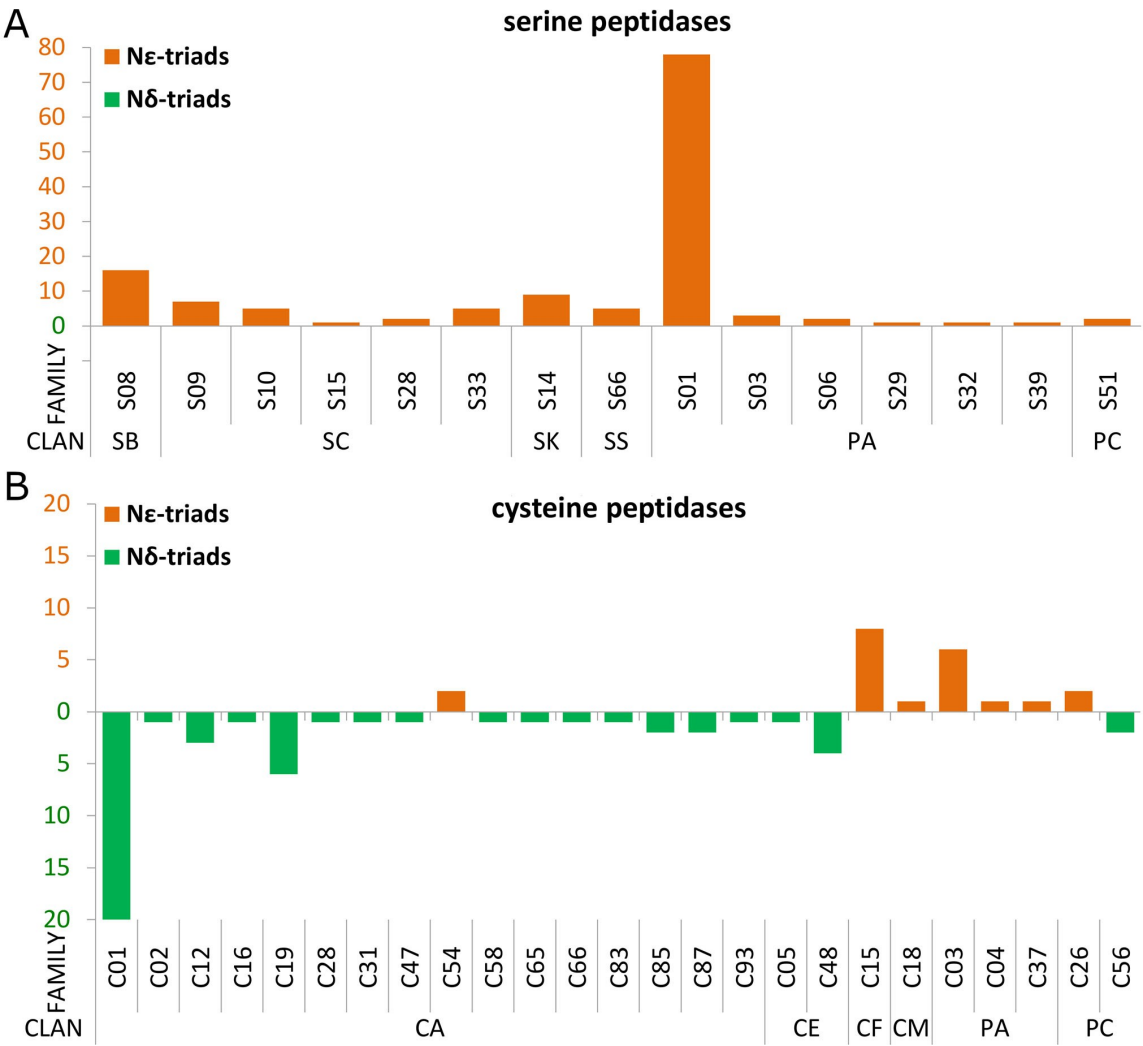
Distribution of Nϵ‐ and Nδ‐triads among A) serine and B) cysteine peptidase families. The cases were weighted based on sequence identity.

Serine peptidase triads are almost exclusively Nϵ‐configured (Figure 2A). The few exceptions are all highly suspicious (Table S2). The unusual histidine orientations result from low resolution of diffraction data, misinterpretation of the electron density or presence of non‐catalytic metal ions in the active sites. Some triads were captured in non‐catalytic conformation as judged from the distances between the triad members, e.g. in ClpP1 of family S14[^16^, ^17^, ^18^] (Figure S5A). The triad classification was ambiguous for mitochondrial serine protease HtrA2 of family S1[^19^, ^20^] (Figure S5B). The Nϵ‐triad configuration has evolved independently in at least six serine peptidase clans, which would occur by chance only in 0.5^6^≈1.6 % of cases (Figure S6A). We conclude that the Nϵ‐triad configuration is likely selected for in serine peptidases.

Cysteine peptidase triads are either Nδ‐ or Nϵ‐configured (Figure S6B). Triad configuration is conserved at the MEROPS family level (Figure 2B). Nδ‐triads were exclusively found in clan CE, Nϵ‐triads in clans CF, CM and PA. Although clans are defined as evolutionarily related, triad configuration is not always conserved at the clan level. Most families within clan CA have Nδ‐triads, but the family C54 (autophagin) is consistently Nϵ‐configured (Figure S5C). Interestingly, the sequence positions of the cysteine and the third triad residue were evolutionarily preserved, whereas the histidine location was not, which resulted in two alternative triad configurations (Figure 3).

**Figure 3:**
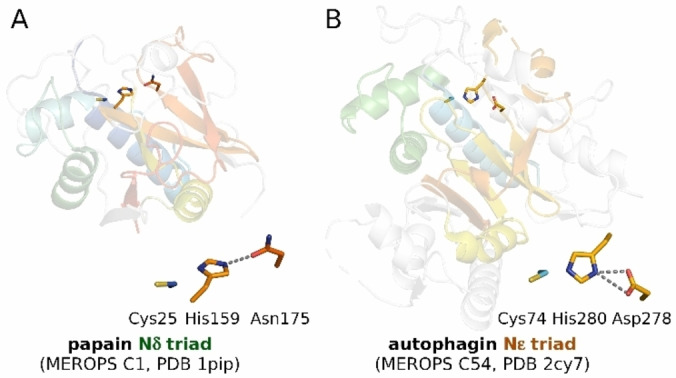
Clan CA peptidases contain both Nδ‐ and Nϵ‐triads: A) papain,^[11]^ B) autophagin Atg4B.^[21]^ The proteins are rainbow‐colored blue to red from the N‐ to the C‐terminus, but only the key corresponding structural elements are shown in color.

In the mixed clan PC, peptidases of the family C26 (γ‐glutamyl‐hydrolase) are Nϵ‐configured, whereas the family C56 enzymes (PfpI) are Nδ‐configured. The S51 serine protease family of the same clan is typically Nϵ‐configured. The location of the catalytic machineries is distinct in the three families except for the Cys/Ser nucleophile (Figure 4). Apparently, evolutionary transitions between the Nδ‐ and Nϵ‐triads involve changes of the triad residue anchoring positions.

**Figure 4:**
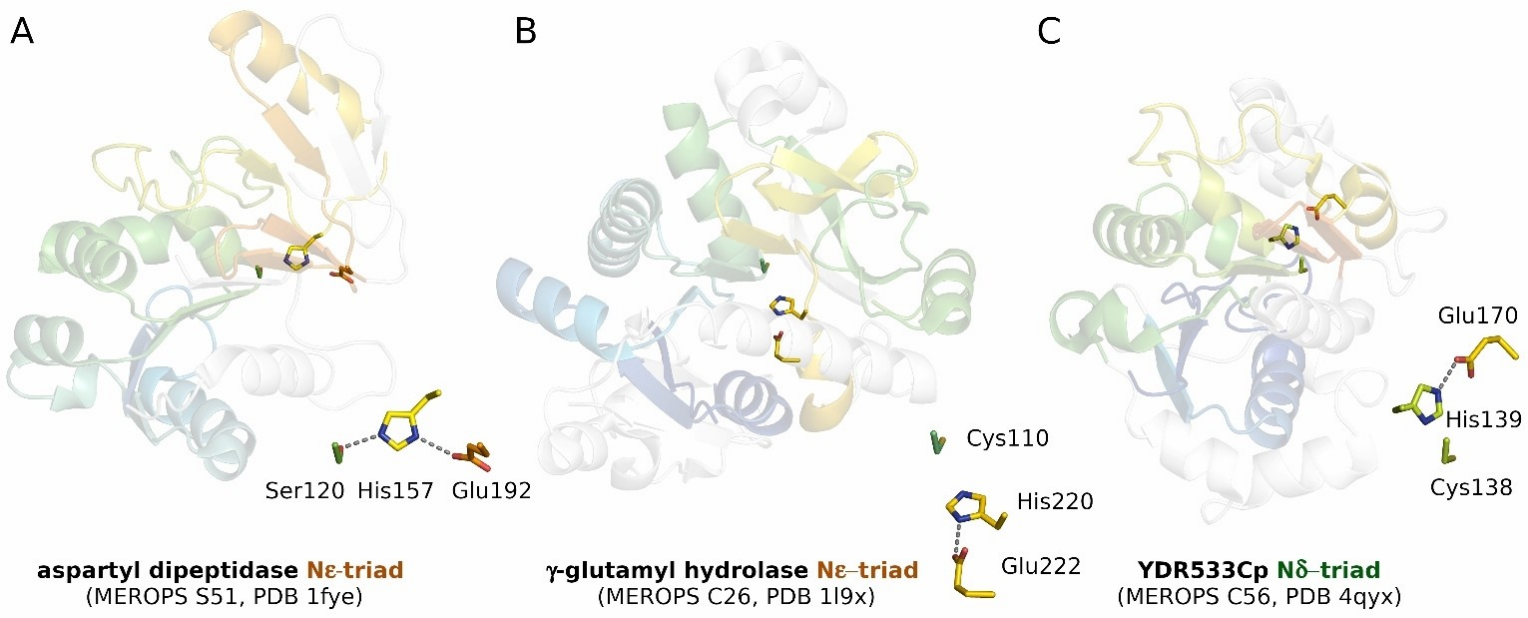
Mixed peptidase clan PC contains Nϵ‐ and Nδ‐triads: A) aspartyl dipeptidase,^[27]^ B) γ‐glutamyl hydrolase,^[28]^ C) YDR533C protein.^[29]^

Our finding that serine, but not cysteine peptidase catalytic triads are exclusively Nϵ‐configured prompted the question whether the rule applies also to non‐peptidases. To test this, we retrieved prototypes of enzymes using Ser/Cys‐His dyads from M‐CSA^[22]^ and EzCatDB.^[23]^ Since the distinction between triads and dyads is often ambiguous (especially for Cys nucleophiles), dyads were used in the scan to achieve maximum possible completeness.

The majority of the collected serine catalytic dyads and triads performed esterase reactions. Further serine triads were found in the active sites of oxidoreductases, transferases, lyases, and isomerases (Table S3A). Despite the wide variety of catalyzed reaction types, the serine residue has a similar mechanistic role in most cases. For the possible exception, hydroxynitrile lyase, a conventional peptidase‐like mechanism with covalent acyl‐serine intermediate,^[24]^ and an alternative mechanism with abstraction of a proton from the substrate[^25^, ^26^] have been proposed.

We next checked the structures of esterases, other α/β‐fold hydrolases and representative enzymes of further catalytic classes for triad configuration. We manually verified 32 classes of enzymes with serine triads and computationally screened 3379 cases (Suppl. Meth.). In all but three distinct enzymes, the catalytic triad was Nϵ‐configured as in serine peptidases (Table S3A, Figure S6C, S7–S8).

The Nδ‐configured exceptions with a serine residue in the triad were the phosphoglucomutases (PGM), 3‐mercaptopyruvate sulfurtransferase (MST) and methylesterase CheB. In PGM, the resting state of the enzyme has a pre‐phosphorylated active site serine, which is dephosphorylated by the attack of a substrate hydroxyl and re‐phosphorylated by the attack on a substrate phosphate^[30]^ (Figure 5A, S9). In MST, a cysteine acts as the nucleophile and a Ser‐His‐Asp triad only facilitates the nucleophilic attack.^[31]^ In CheB, the triad is clearly Nδ‐configured, as already noted by the structure authors^[32]^ (Figure 5B). Mutagenesis data and conservation of the CheB triad residues support their role in catalysis, despite the insensitivity of the enzyme to DFP, a potent inhibitor of serine hydrolases.^[33]^

**Figure 5:**
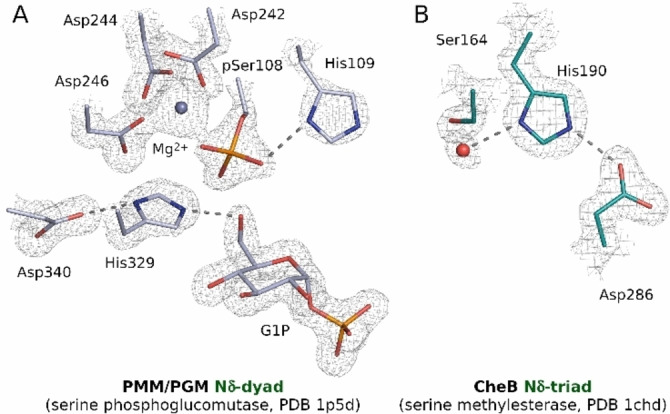
Unusual enzymes with a catalytic serine Nδ‐triad/dyad: A) phosphoglucomutase,^[34]^ B) CheB methylesterase.^[32]^ 2*F*_o_‐*F*_c_ density maps are contoured at 1.5 rmsd.

Catalytic cysteine triads in non‐peptidase enzymes are rarer than the serine ones. In many cases, the Cys‐His dyad is sufficient to perform the reaction. The histidine is often observed in alternative orientations in the structures of similar enzymes. The enzymes with cysteine catalytic triads perform a large variety of reactions covering almost all EC classes. Despite this variability, the triads are mostly Nϵ‐configured. The Nδ‐configured triads were found only in transferases (Table S3B).

The near absence of Nδ‐configured serine catalytic triads suggests a catalytic disadvantage. To estimate the magnitude of this effect, we scanned the literature for the reports of engineered Ser/Cys nucleophile substitutions (Table S4). Typically, such changes are detrimental to catalytic activity. However, substitutions in the context of Nϵ‐triads sometimes have moderate effects. Switches of catalytic activity[^35^, ^36^, ^37^, ^38^] or even increased activity (in combination with supporting mutations) have been reported.[^39^, ^40^] Cys→Ser changes in the Nδ‐configuration abolished activity throughout, as the Nϵ‐rule would suggest.

Next, we analyzed nucleophile switches that have occurred in the course of evolution (Table S5). For all cysteine (TGT, TGC) and some serine (AGT, AGC, TCC, TCT) codons, such exchanges are possible with single base substitutions. There are convincing examples of likely evolutionary Ser/Cys switches in the context of Nϵ‐triads. Chymotrypsin family enzymes share the same fold and active site location with 3C proteases (Figure S10).[^41^, ^42^] Both Ser and Cys residues in the nucleophile position are found in thioesterases,^[43]^ N‐acyltransferases^[35]^ and dienelactone hydrolases^[44]^ of α/β‐hydrolase fold. Cysteine nucleophiles are also found in a few further groups of α/β‐hydrolases with preserved triad residue locations (Figure S11). Interestingly, the groups of enzymes for which serine and cysteine nucleophiles exist in nature appear to respond better to *in vitro* nucleophile substitutions.

Surprisingly, Ser↔Cys exchanges seem to occur naturally also in the context of Nδ‐triads. Such a switch with limited effect on catalytic activity has been reported for the following four enzymes related to the C1 family of cysteine peptidases (CA clan). For the SERA5 protein from *P*. *falciparum*, reports on the activity of the serine variant are conflicting.[^45^, ^46^] In this enzyme, and also in congopain from *T. congolense* ^[47]^ and EnCL from *E. nipponicum*,^[48]^ the Cys→Ser nucleophile switch is likely an adaptation of the hijacked enzymes to the parasite lifestyle and higher exposure to oxidative stress. In silicateins, a Cys→Ser exchange is accompanied by a switch to siloxane polymerization activity with limited or no proteolytic activity preserved.[^49^, ^50^] We conclude that Nδ‐triads with serine nucleophile can occur naturally, albeit extremely rarely.

The preference for the Nϵ‐configuration in catalytic triads with a serine, but not cysteine nucleophile, may be understood mechanistically. For serine triads, the proton transfer in the initiating step of catalysis is against a strong p*K*_a_ gradient and can only take place during catalysis, when the serine is “acidified” by proximity to the substrate carbonyl carbon. As a result, the active site geometry is much more optimized and constrained in serine than cysteine triads (the nucleophile and the third triad residue hydrogen bond acceptor are closer to the idealized positions with respect to the histidine, Figure S2),^[51]^ despite similar B‐factor distributions and Ramachandran plots (Figure S12–S17). In enzymes with a cysteine nucleophile, a third triad residue can even be altogether missing.

The necessity for the proton transfer during the reaction and a shorter van der Waals radius of oxygen than sulfur (1.52 versus 1.8 Å) cause greater proximity of serine than cysteine triads to the substrate. Upon a computational Nϵ and Nδ swap, this proximity causes steric conflict with the Cβ atom of the catalytic histidine (Figure S18). Serine triads with their more rigid architecture may be more sensitive to such conflict and the resulting distortions than cysteine triads.

As the Nϵ‐rule applies to almost all known serine triads, arguments based on substrate specific features cannot be a full explanation. Serine catalytic triads have almost universally aspartate or glutamate as the third triad residue (Figure 6, S19–S20). As contact to the negatively charged residue will raise histidine p*K*_a_ more than for the neutral asparagine or glutamine, the highly non‐random choice suggests evolutionary pressure to optimize the proton transfer step.

**Figure 6:**
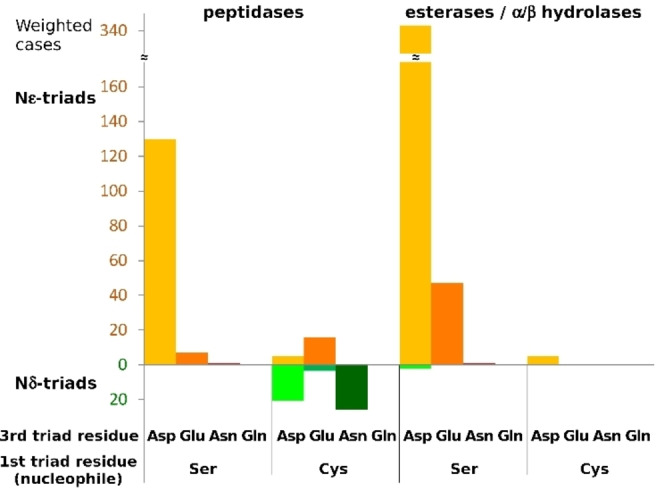
The identity of the third triad residue in Nϵ‐ and Nδ‐triads. The cases were weighted based on their sequence identity. There is a single case of a Cif type III effector with glutamine as a third triad residue,^[52]^ but its peptidase activity was not detected and thus it is not included in our set.

Since the histidine Nϵ is more basic than its Nδ, the Nϵ‐triad configuration optimizes the “difficult” proton transfer step, which is likely to be rate‐limiting for serine, but not cysteine triads. We hypothesize that the resulting catalytic advantage for serine nucleophiles is sufficient for its almost universal selection, even though it comes at a cost for protein stability. For cysteine nucleophiles, the p*K*_a_ gradient for the proton transfer is smaller, and it is not expected to be rate limiting. Consequently, its facilitation is not as critical, explaining the presence of both Nδ‐ and Nϵ‐triads.

In closing, we note that our interpretation of the Nϵ‐rule for serine catalytic triads fits into the broader concept of “frustration in biomolecules”.^[53]^ It has been suggested that suboptimal selection of amino acids (mutational frustration) and interactions (configurational frustration) is enriched in the vicinity of active sites.^[54]^ It is thought that these energetically unfavorable choices persist in evolution because they confer catalytic advantage. In this work, we have revealed the almost universal choice of the Nϵ‐configuration for catalytic triads with serine nucleophiles in the active sites. The configuration appears to be selected for catalytic advantage, despite the stability cost, and may therefore be a form of configurational frustration.

## Conflict of interest

The authors declare no conflict of interest.

## Supporting information

As a service to our authors and readers, this journal provides supporting information supplied by the authors. Such materials are peer reviewed and may be re‐organized for online delivery, but are not copy‐edited or typeset. Technical support issues arising from supporting information (other than missing files) should be addressed to the authors.

Supporting Information

## Data Availability

Data sharing is not applicable to this article as no new data were created or analyzed in this study.
