# Supplementary material for: The Nϵ‐Rule for Serine, but Not Cysteine Catalytic Triads
Source: Angew Chem Int Ed Engl. 2022 Sep 5;61(42):e202206945. doi: 10.1002/anie.202206945 (PMC9825947; doi:10.1002/anie.202206945)
Supplement: Supplementary file 1 — Supporting Information [file ANIE-61-0-s001.pdf]

Angewandte Chemie
Eine Zeitschrift der Gesellschaft Deutscher Chemiker

Supporting Information

# The Nε-Rule for Serine, but Not Cysteine Catalytic Triads

*H. Czapinska, M. Bochtler**

# Supplementary Material

## Supplementary Methods

### Triad search procedure

Triads were automatically selected based on geometry. Approximate locations of the nucleophile Oγ/Sγ and the hydrogen bond acceptor oxygen in the third triad residue relative to the imidazole ring of the histidine can be deduced from first principles. For simplicity, this is presented here in detail only for an Nε-configured triad (Fig. S1). An analogous argument can be made for an Nδ-configured triad. The proton that is transferred to the histidine during or prior to the reaction can also be considered as part of a hydrogen bond. This hydrogen bond is optimal when the proton approaches the unoccupied $sp^2$ lone pair of the nitrogen imidazole. Neutron diffraction data confirm this proton location[1]. Assuming the optimal hydrogen bond donor-hydrogen-acceptor angle close to 180° (for typical deviations, see Wood et al.[2]), the Oγ/Sγ should be located in the plane of the histidine imidazole ring. The imaginary line running through the nucleophile and the Nε histidine atom should run through the midpoint of the histidine Nδ and Cγ bond. A similar argument can be made to deduce the optimal location for the oxygen atom of the third triad residue. For the Nδ-configured triad, the nucleophile should also lie in the imidazole plane, on a line passing through Nδ and a midpoint of the histidine Cδ and Nε bond.

We used the histidine residues as a search start, and then determined the coordinates of the optimal hydrogen bonding partner locations 2.8 Å away from the Nδ/Nε atom. We searched for atoms in spheres centered at these positions and selected all histidines that had a serine Oγ or cysteine Sγ atom within one of the spheres and an acidic/amide side group of aspartate, glutamate, asparagine or glutamine within the other (Fig. S1).

All available 3D structures were downloaded from the Protein Data Bank (PDB)[3] on 14.07.2021. The structures determined by NMR were eliminated. The histidines with missing atoms were filtered out. The modified amino acids such as thio- or oxycysteines were excluded from the search. The triads were partitioned into Nδ- and Nε-configured groups. Next, we downloaded from the PDB amino acid sequences for protein chains that contained selected histidine residues. We clustered the obtained sequences with CD-HIT[4] using a sequence identity of 0.7 as a cut-off. We tried several different cutoffs and word lengths and used the most generous one that worked stably for all cases. We weighted the triad occurrences based on the cluster size. Multiple instances in the same PDB entry (two or more triads) or multiple identical protein copies in the asymmetric unit of the crystal were counted as independent in the clustering procedure.

**Non catalytic triads**

The search for triads was performed according to the above procedure. To avoid high rate of false positives, we used narrowly defined sphere around the hydrogen bond partner locations (3 Å radius). We have found 109810 putative triads. To assess the configuration of non-catalytic triads we filtered out all PDB entries corresponding to peptidases and esterases based on the EC numbers in the PDB entries, text search of the files (inhibitors excluded) and presence in the MEROPS[5] and ESTHER[6] databases. Altogether 86176 triads were kept in the control non-catalytic set and 23634 triads were filtered out. The triad statistics are presented in Tab. S1.

**Peptidases**

We relied on MEROPS[5] as a source of information on the peptidase structures. We downloaded all structures listed in MEROPS from the PDB. We corrected entries that were annotated in duplicate or misannotated in MEROPS due to the presence of multiple peptidases in a single polypeptide chain, which resulted in 3236 serine and 1078 cysteine peptidase structures. The structures containing non-catalytic domains were filtered out at the triad search step. The dyad containing peptidases were excluded from the analysis. Due to the conformational perturbations introduced e.g., by the presence of inhibitors, the search performed with the stringent 3 Å radius missed multiple entries, in particular for cysteine peptidases. Therefore, we used more generous 4 Å search radius and manually curated the obtained set of triads based on sequence numbers of catalytic histidine residues and visual inspection. The final set contained 3187 serine peptidase triads and 593 cysteine peptidase triads. Within serine peptidases we found 3187 Nε-triads and no Nδ-triads and within cysteine peptidases 144 Nε-triads and 449 Nδ-triads. After the CD-HIT clustering performed within MEROPS families/clans we obtained 138 serine triad cases (all Nε) and 71 cysteine triad cases (21 Nε and 50 Nδ cases).

**Esterases / α/β-fold hydrolases**

To our knowledge there is no esterase repository that is as comprehensive as MEROPS. We downloaded all PDB entries that were listed in the ESTHER[6] database of α/β-fold hydrolases. We additionally downloaded all entries in the PDB that were defined by the 3.1 EC class. Moreover, we verified the completeness of the combined set of structures by downloading the PDB files listed in CAZY[7], ThYme[8] and LED[9] databases. In total 4370 structures were screened for the presence of catalytic triads. The final set contained 3365 serine triads and 14 cysteine triads. Within serine enzymes we found 3356 Nε-triads and 9 Nδ-triads and within cysteine enzymes 14 Nε-triads and no Nδ-triads. We verified that all triad containing groups of enzymes from the three databases are represented in our combined set. Next, we created the cross-correlation table of all databases in line with the tables presented by the ESTHER authors[6] (Tab. S6).

All entries that were not listed in ESTHER were stored as a separate group. We performed the search for triads analogously as described for the peptidases and clustered the cases with CD-HIT using 0.7 cut-off. The ESTHER entries were clustered within the families and the remaining entries were clustered separately. We merged the clusters of the remaining entries with the ESTHER ones based on the UNIPROT accession IDs[10] and verified that according to the PFAM[11] classification the clusters in the two groups do not overlap (provided that the PDB entry contained a UNIPROT ID and the UNIPROT entry contained a PFAM ID). We clustered all entries based on their PFAM identifiers and kept the entries that were not PFAM annotated as a separate group. We did not eliminate the peptidases and other groups of non-esterases that were included in the ESTHER database from the set to maximize completeness. After the CD-HIT clustering we obtained 450 serine triad cases (448 Nε and 2 Nδ cases) and 8 cysteine triad cases (all Nε), but some cases could be counted twice because of missing PFAM annotations. Within the PFAM annotated group we collected 393 serine triad cases (391 Nε and 2 Nδ cases) and 5 cysteine triad cases (all Nε).

## Supplementary Figures

**Fig. S1: Design of the search for the putative triads.** The imaginary axes running through the Nε/Nδ histidine atoms and the expected positions of hydrogen bond donors/acceptors in the and first third triad residue are marked with continuous thin lines. The idealized locations of hydrogen bond donors/acceptors are shown as red crosses (sphere centers). The search radius (r) of 3 Å was used for non-catalytic triads and of 4 Å for catalytic triads.

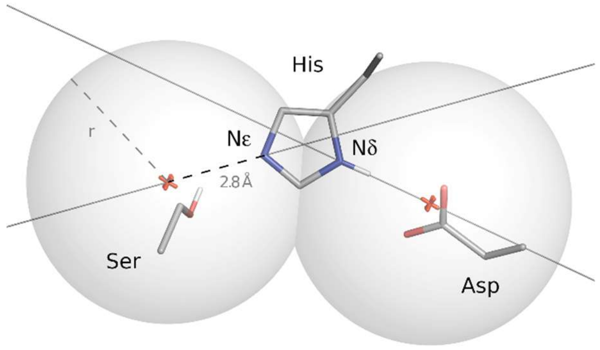


**Fig. S2: Dependence of true and false positive triad cases on the applied search radius.** The search for serine and cysteine peptidase triads performed with different search radii (r) yielded the depicted fractions of **(A)** catalytic triads, **(B)** non-catalytic "triads", constituting predominantly search method artifacts (located in the parts of the search sphere that do not correspond to the proper triad arrangement). The numbers of triads were normalized with respect to the numbers found with 4 Å radius. Please note that serine triads reach near completeness with significantly smaller search sphere. Panel (B) reflects the growing number of artifacts that limits further radius increase.

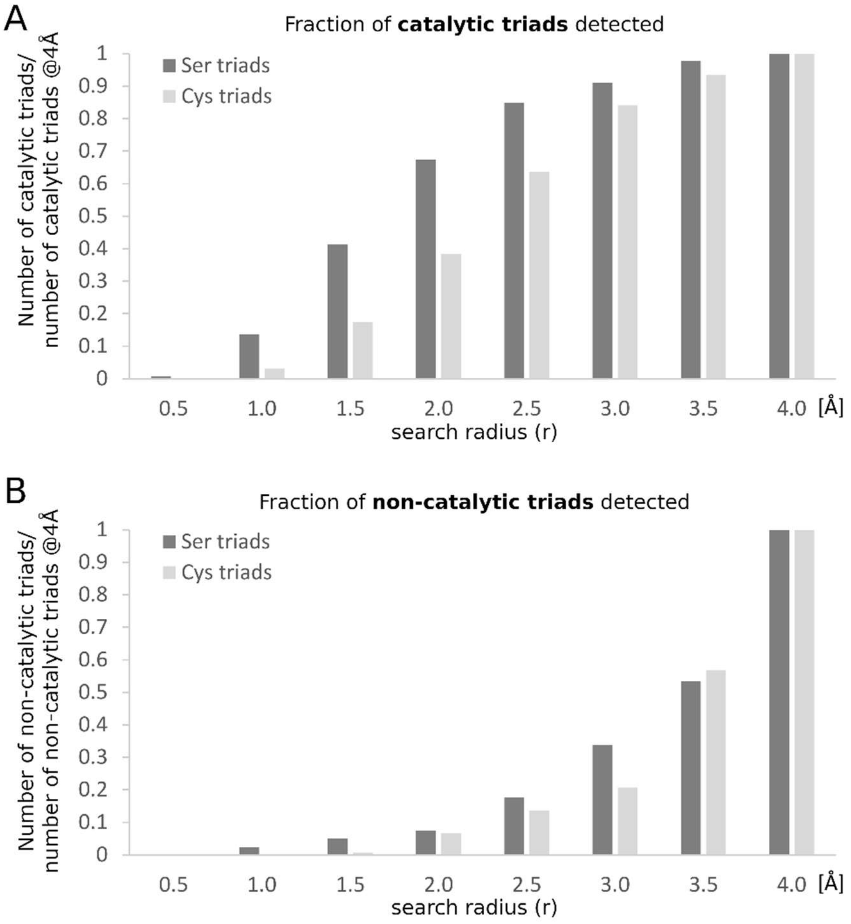

**Fig. S3: Distribution of Nε- and Nδ-triads among (A) serine and (B) cysteine peptidase families.** The cases were <u>not weighted</u> and thus the distribution is skewed towards more heavily investigated enzymes and the ones with multiple protomers in the asymmetric units of the crystals.

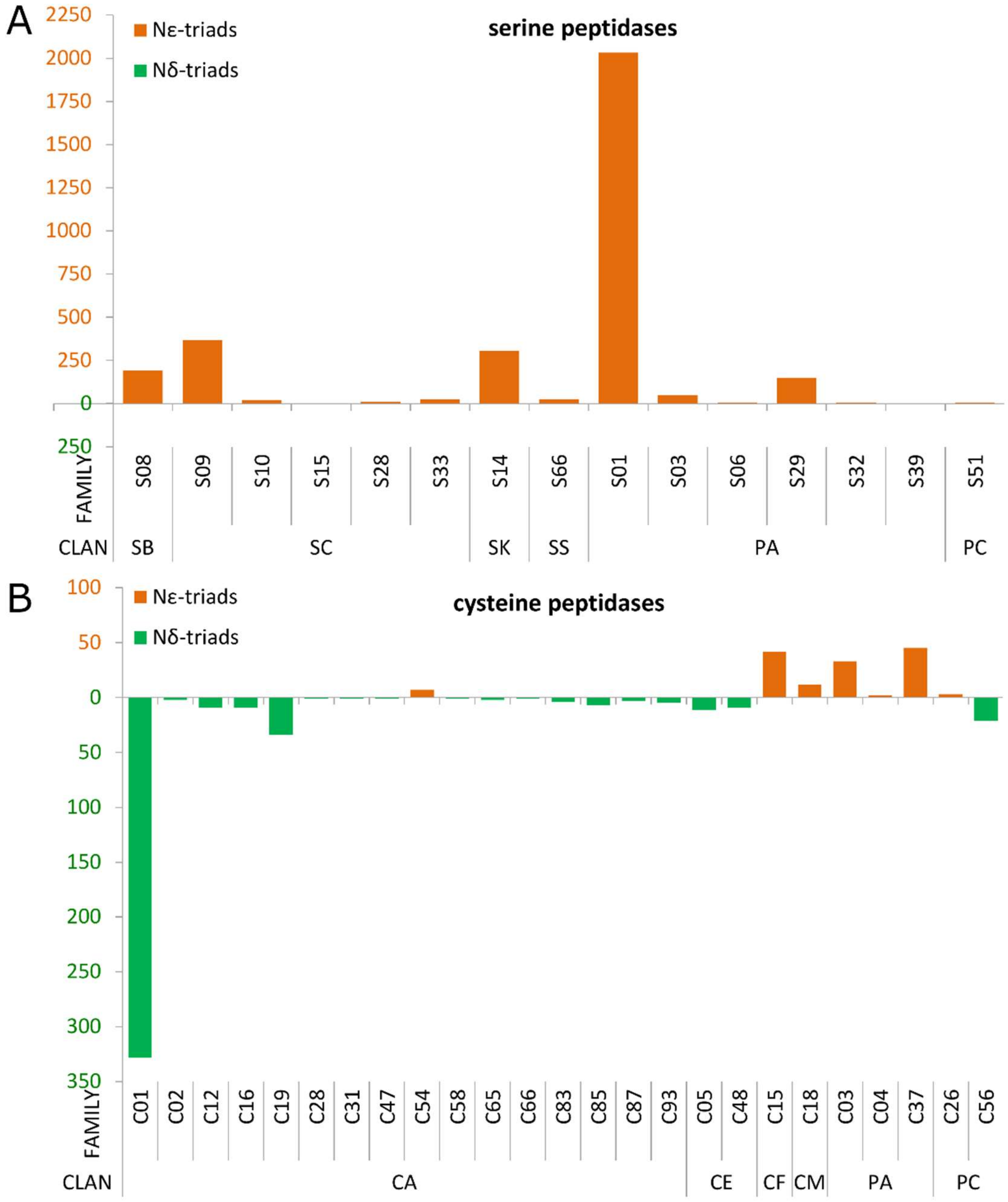

**Fig. S4: Distribution of Nε- and Nδ-triads among (A) serine and (B) cysteine peptidase clans.** The cases were weighted based on their protein sequence identity using CD-HIT[4] (with 0.7 cutoff).

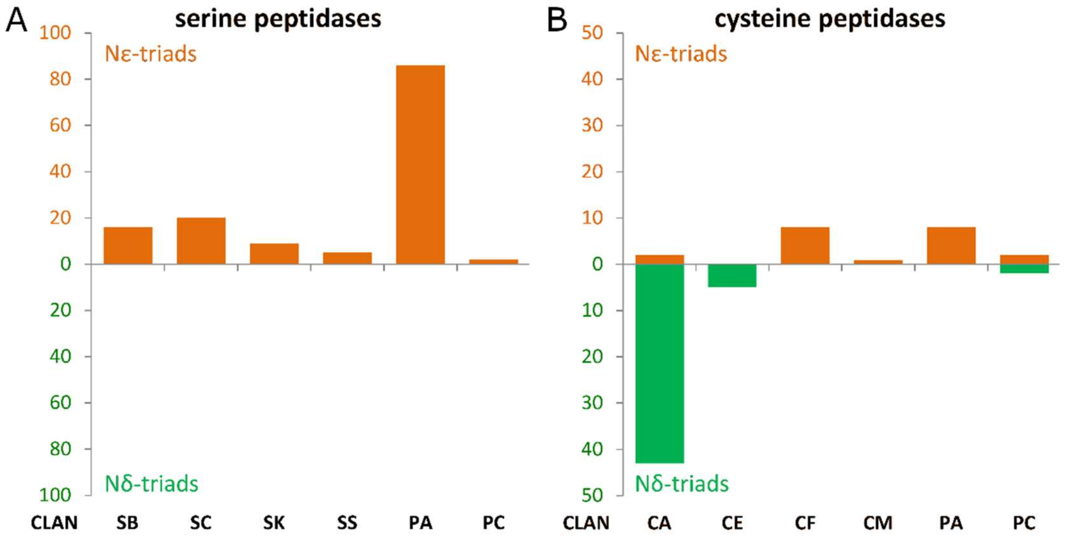


**Fig. S5: Examples of unusual triad conformations among (A, B) serine and (C) cysteine peptidases. (A)** The catalytic triad of ClpP1 was captured in inactive conformation with distances between the triad members exceeding typical hydrogen bond lengths[12]. **(B)** The triad conformation in the mitochondrial serine protease HtrA2 structure is ambiguous due to unclear histidine orientation in which neither Nε nor Nδ clearly points to hydrogen bond partners[13]. **(C)** Autophagin is an outlier among cysteine peptidases of clan CA. Interestingly, its triad is surrounded by multiple alternative hydrogen bond partners[14]. The densities correspond to $2F_o$-$F_c$ maps calculated based on deposited structure factors and contoured at 1.5 rmsd. The residue numbers in (A) and (B) were adopted from the articles and correspond to S91, H116, D163 and E132 in 4jcq and S173, H65 and D95 in 5wyn file

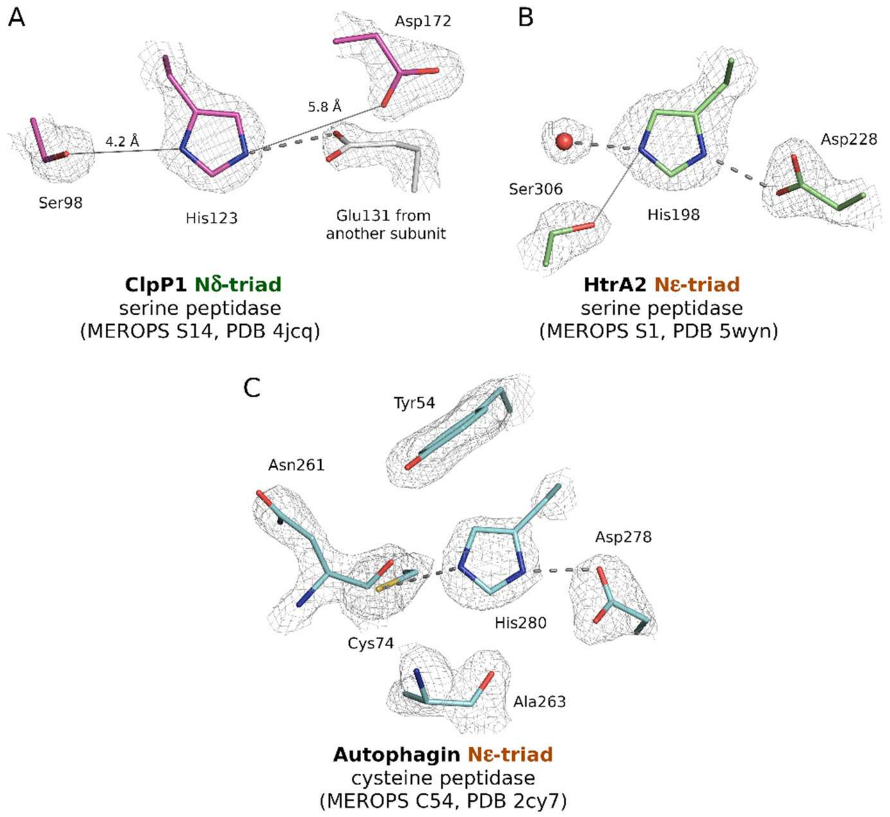

**Fig. S6: Representative examples of Nδ and Nε triad containing (A) serine and (B) cysteine peptidase clans and (C) esterase folds.** Histidine imidazole rings were used to position the enzymes. The active site mutations were reverted. Please note that in contrast to the catalytic nucleophile that usually occupies a position close to the N-terminal end of a helix, the other two triad members are anchored in variable structural elements with inconsistent main chain conformation and directionality.

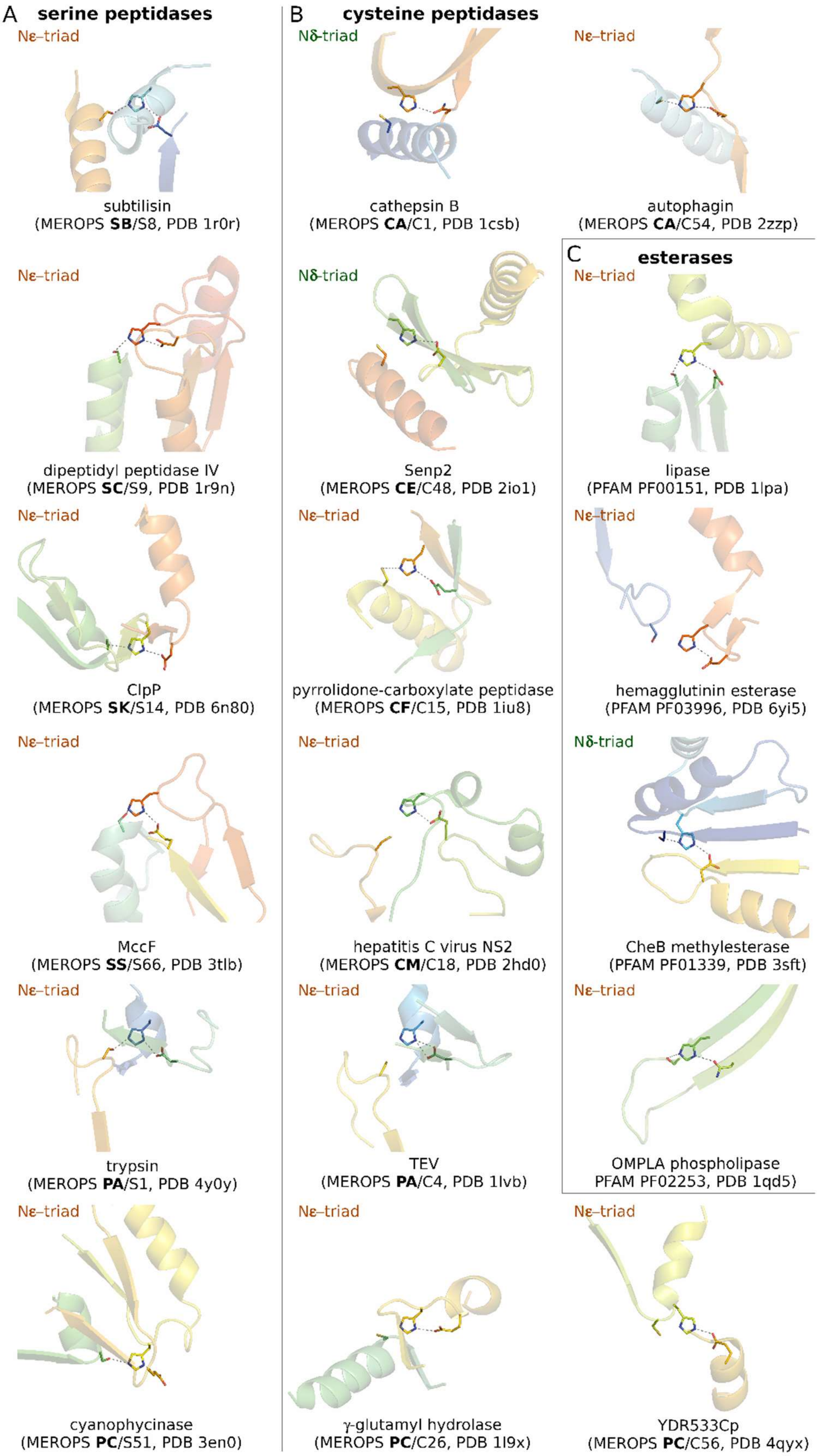

**Fig. S7: Distribution of Nε- and Nδ-triads among (A, B) serine and (C) cysteine esterases / α/β-fold hydrolases.** The cases were not weighted and thus the distribution is skewed towards more heavily investigated enzymes and the ones with multiple protomers in the asymmetric units of the crystals.

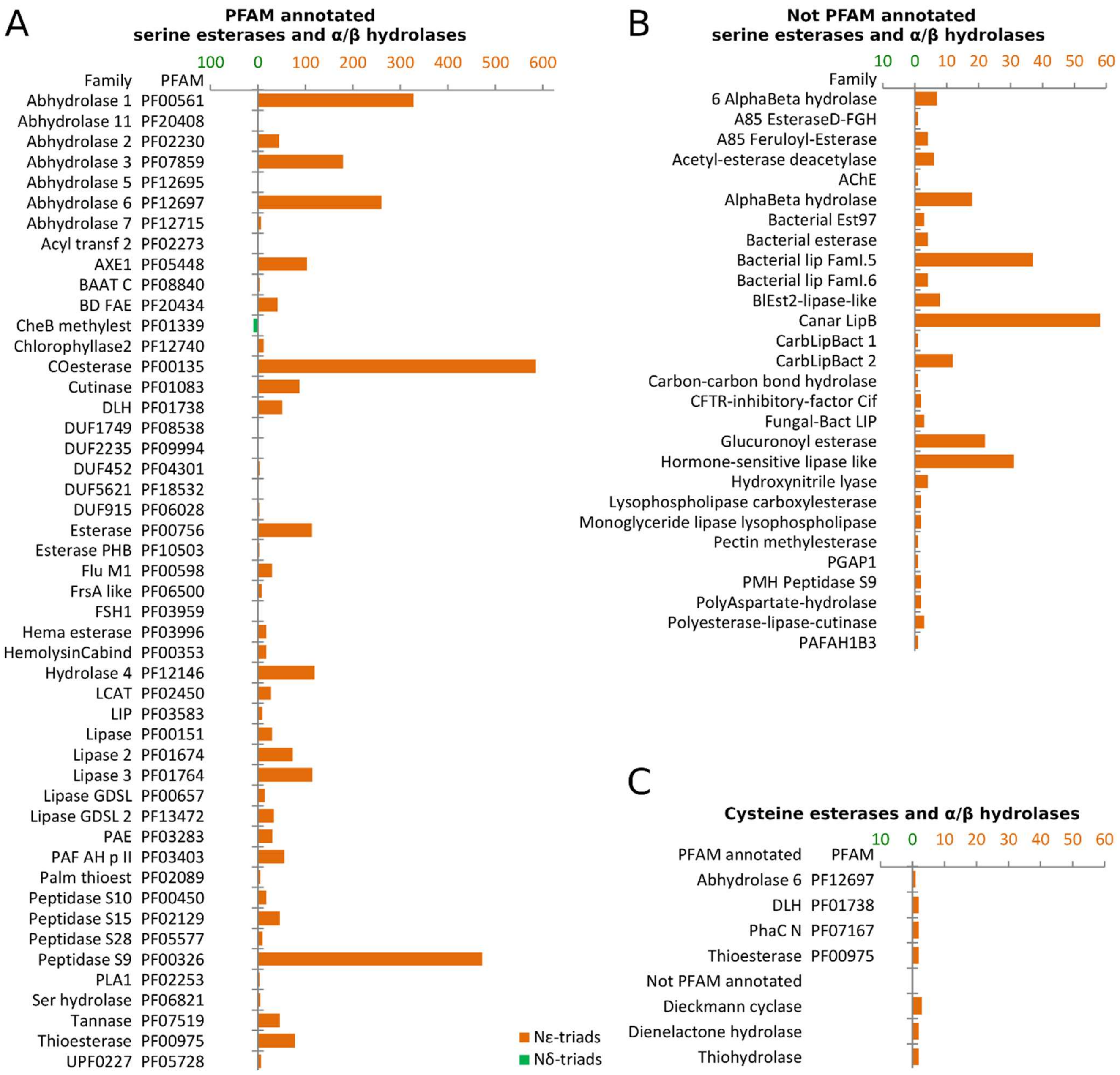

**Fig. S8: Distribution of Nε- and Nδ-triads among (A) serine and (B) cysteine esterases / α/β-fold hydrolases.** The cases were <u>weighted</u> based on their protein sequence identity using CD-HIT[4] (with 0.7 cutoff).

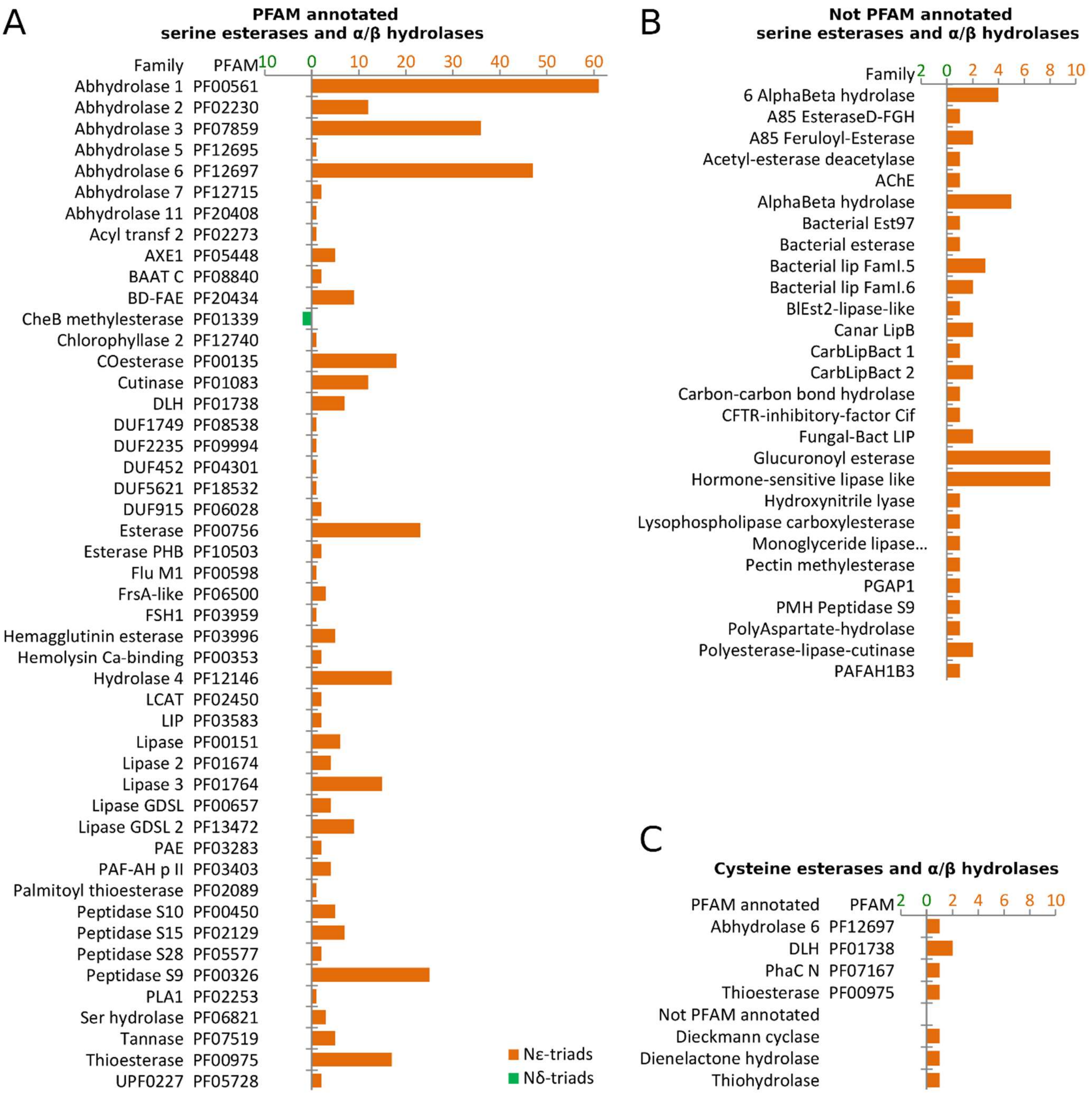

**Fig. S9: Schematic view of the reaction mechanism of phosphoglucomutases selected for the Nδ-configuration of the active site histidine in the His-Ser dyad.** The third triad residue was likely a search procedure artifact and is thus not shown. The reaction mechanism of phosphoglucomutases differs substantially from the one of serine nucleophile employing enzymes since the serine in the state preceding the reaction is phosphorylated.

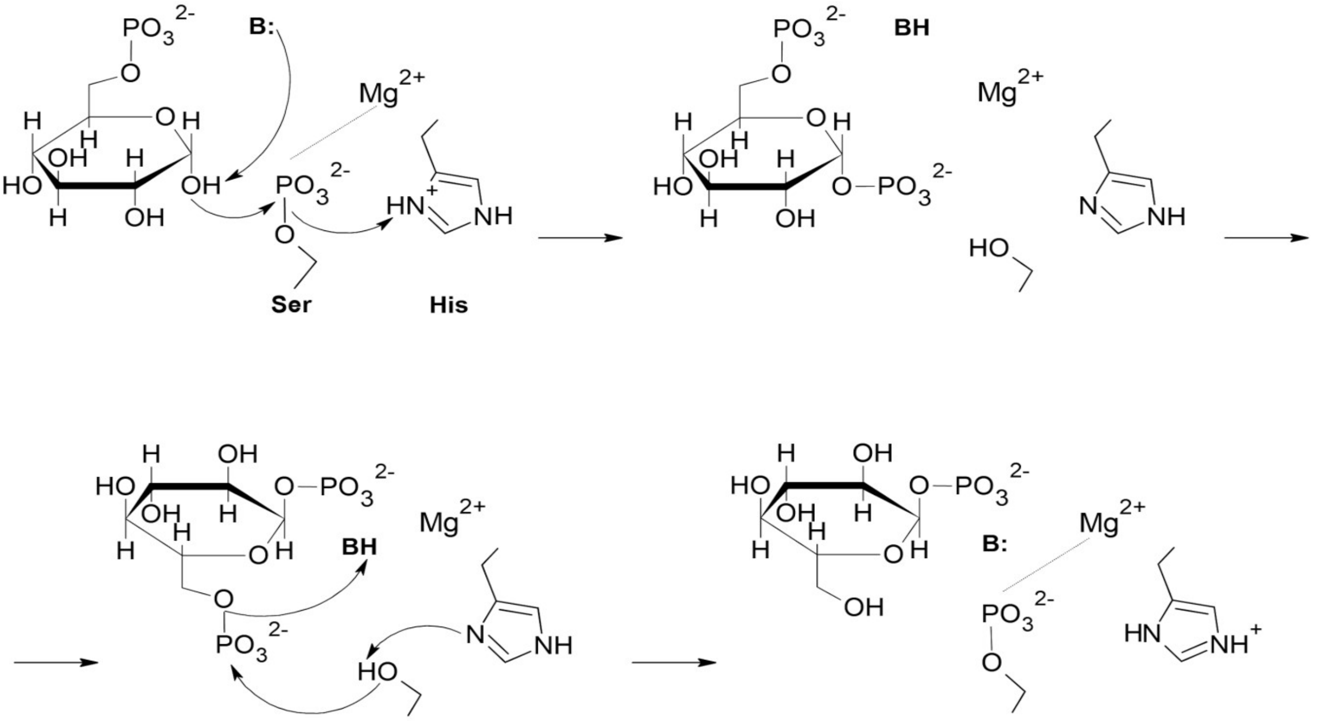


**Fig. S10: Evolutional Cys/Ser nucleophile switch among peptidases. (A)** Chymotrypsin family peptidases (MEROPS family S1, clan PA) contain Ser-His-Asp catalytic triad. **(B)** Viral 3C(-like) peptidases of the same fold (family C3, clan PA) contain Cys-His-Asp/Glu catalytic triads. Trypsin[15] and human rhinovirus 3C protease[16] were used as representatives the two groups**.** The proteins are shown in ribbon representation and rainbow-colored blue to red from N- to C-terminus. The triad residues are indicated and magnified with hydrogen bonds shown as dashed lines (consistent color scheme was used).

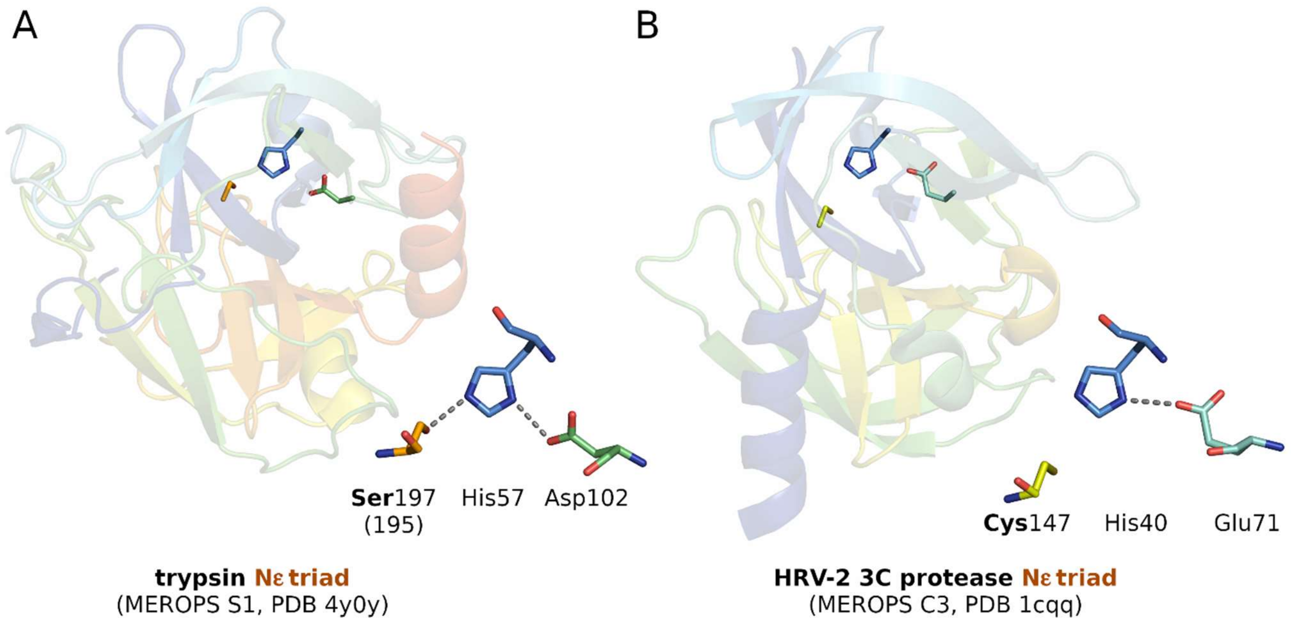

**Fig. S11: Evolutionary Cys/Ser nucleophile switches among α/β-hydrolases.** The residues constituting catalytic cysteine triads present in some α/β-hydrolases occur in analogous places as in the α/β-hydrolases with serine triads (here depicted with prolyl oligopeptidase). Two alternative positions of the third triad residue are observed in both cases. **(A)** prolyl oligopeptidase[17], **(B)** dienelactone hydrolase[18], **(C)** polyhydroxyalkanoate synthase[19], **(D)** GrgF[20], **(E)** Dieckmann cyclase[21], **(F)** nonribosomal peptide synthase[22].

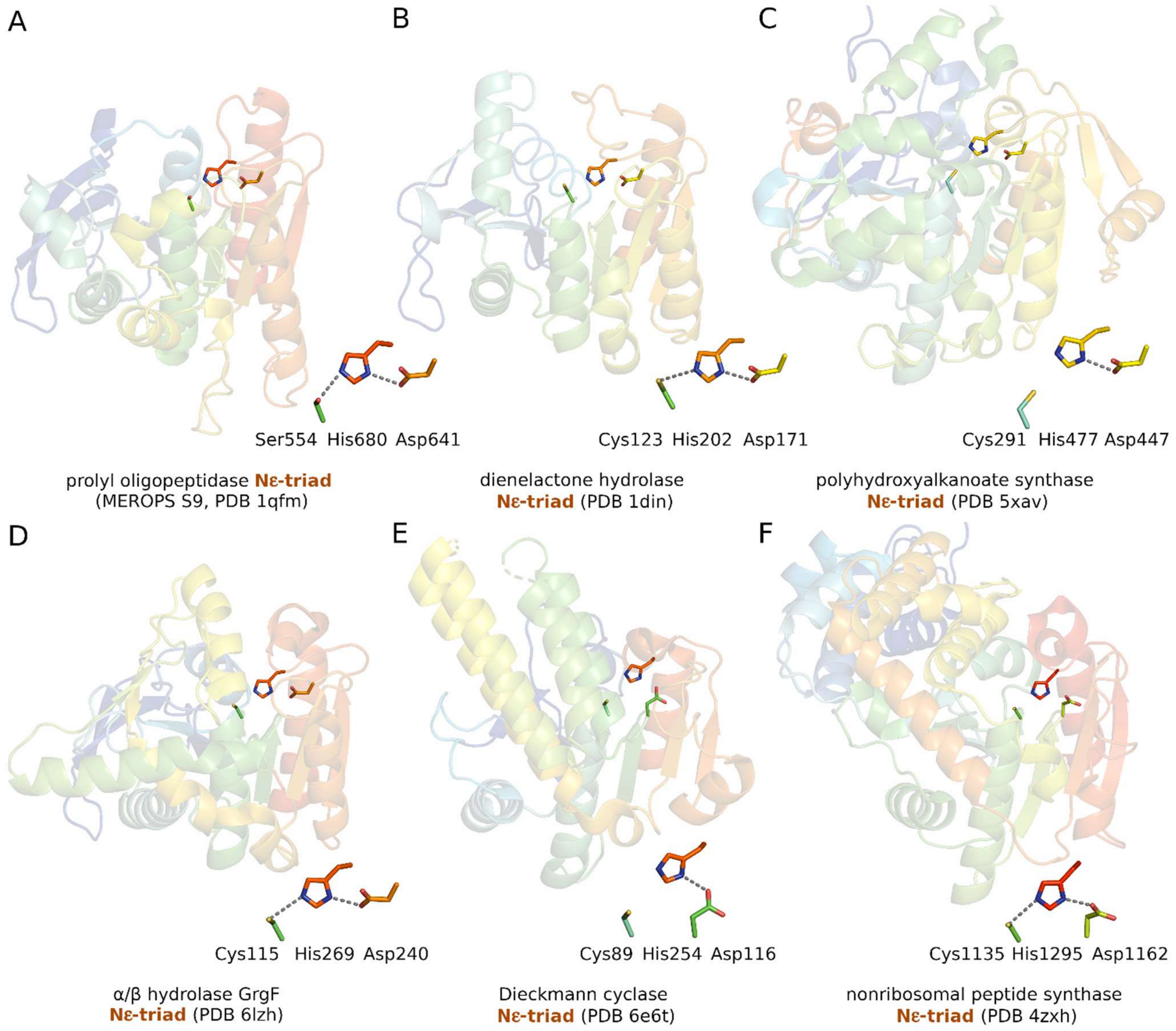

**Fig. S12: Ramachandran plots of control and active site residues in serine peptidases.** The plots were generated with a modified version of Ramachandran-Plotter-v2.0.2 (https://github.com/Joseph-Ellaway/Ramachandran_Plotter) with background empirically generated from the Top8000 PDB dataset. For control panels in Figs. S12-S17 all residues of a given type present in the PDB files containing the studied catalytic triads were used. Residual catalytic triads may be present in the control set, as there could be molecules in the asymmetric unit with triads in inactive conformation accompanying the active ones.

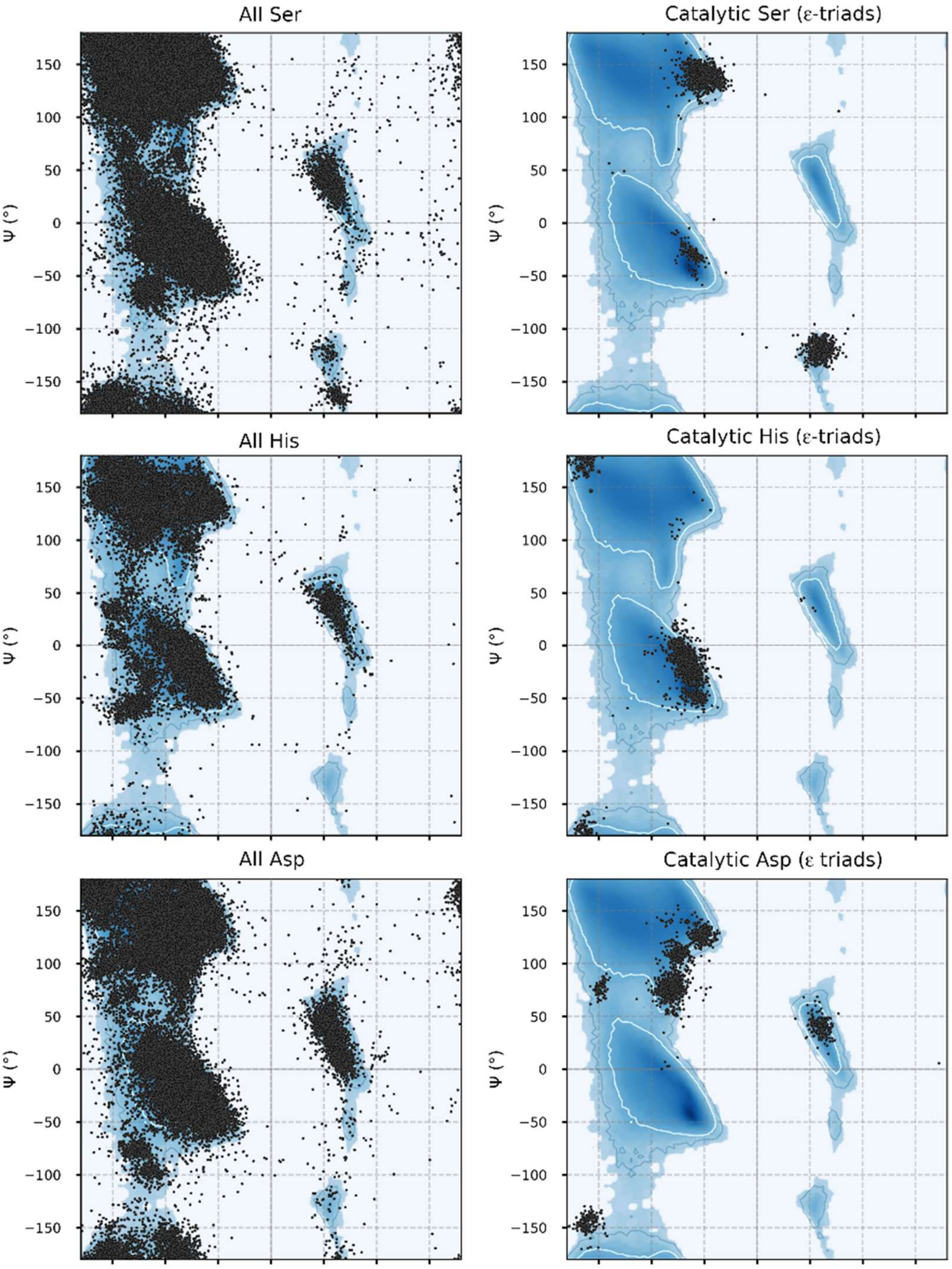

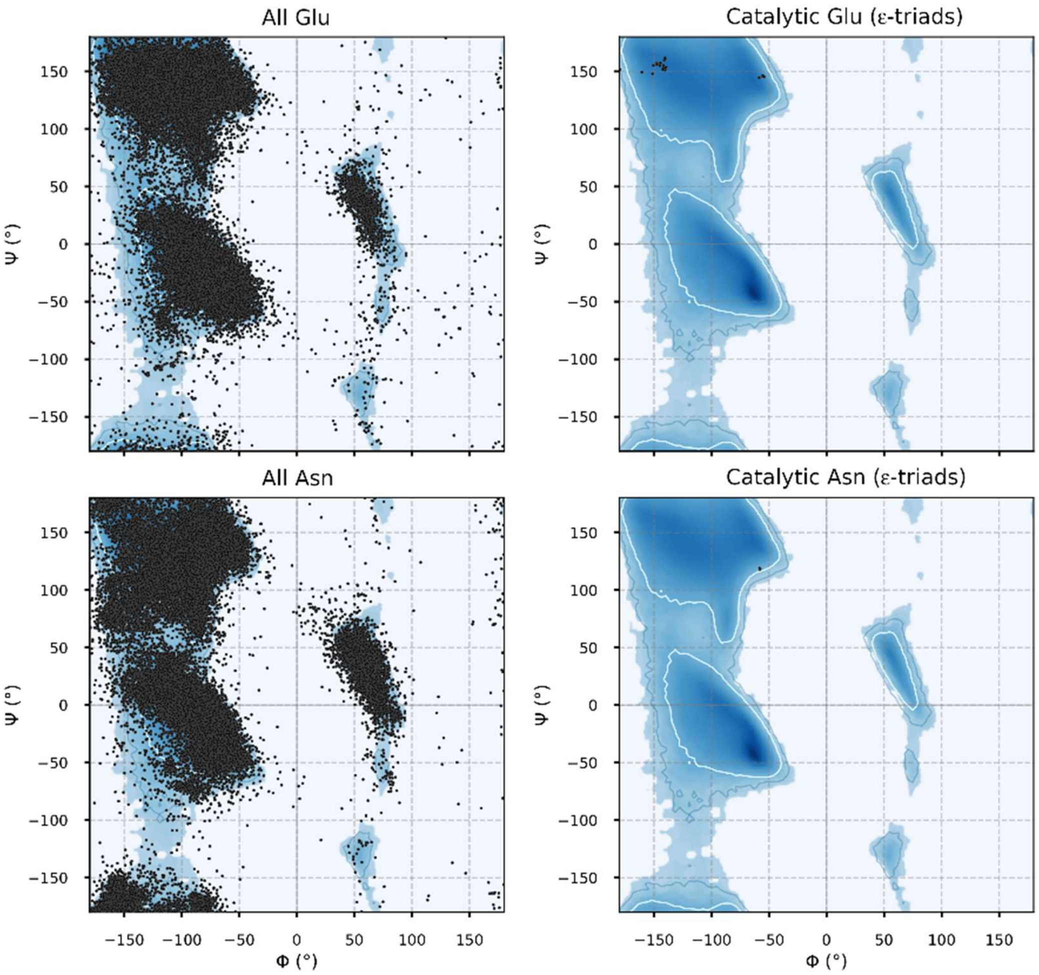
All Glu
Catalytic Glu (ε-triads)
All Asn
Catalytic Asn (ε-triads)
Ψ (°)
Φ (°)
150
100
50
0
−50
−100
−150

**Fig. S13: Ramachandran plots of control and active site residues in cysteine peptidases.**

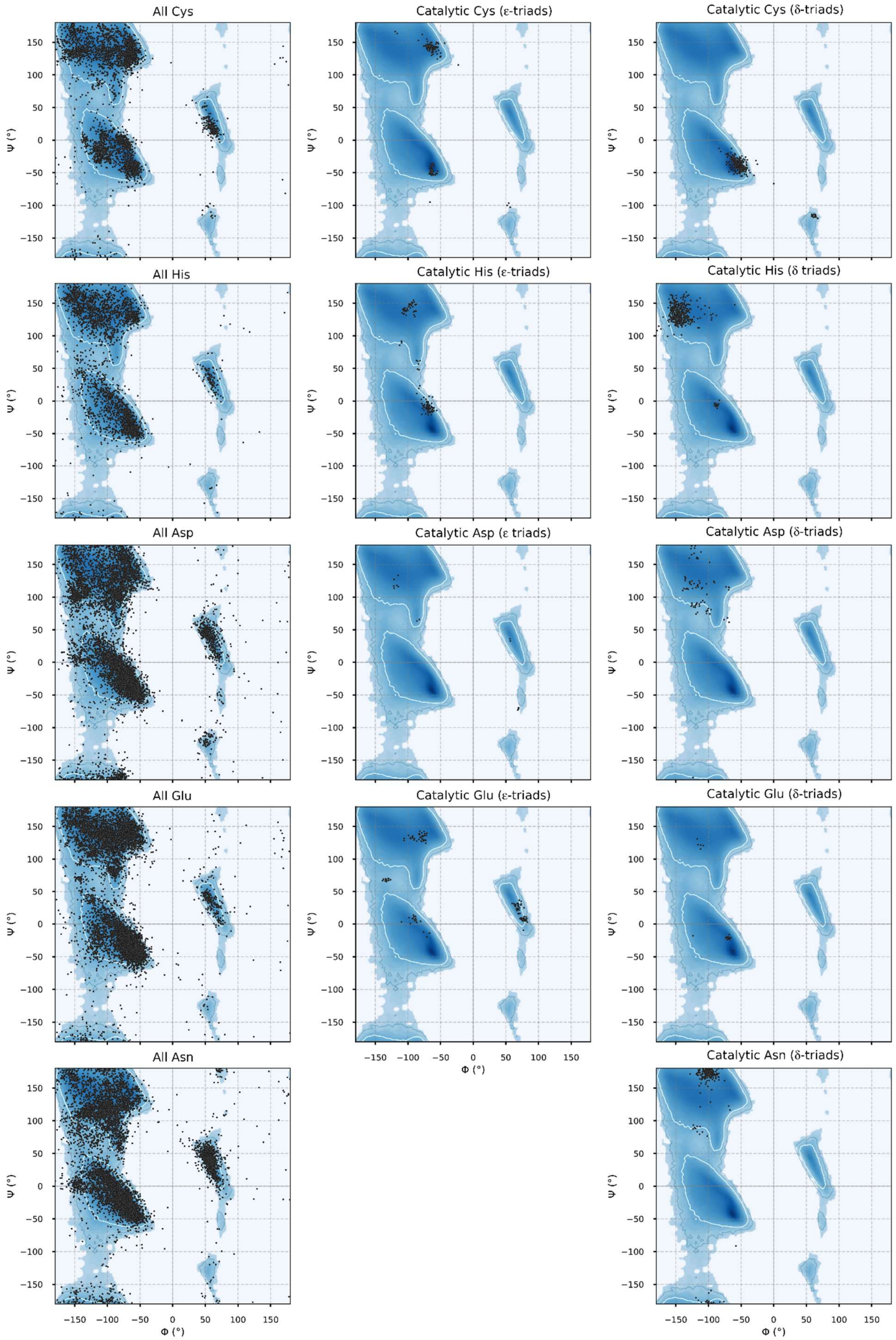

**Fig. S14: Ramachandran plots of control and active site residues in serine esterases / α/β-hydrolases.**

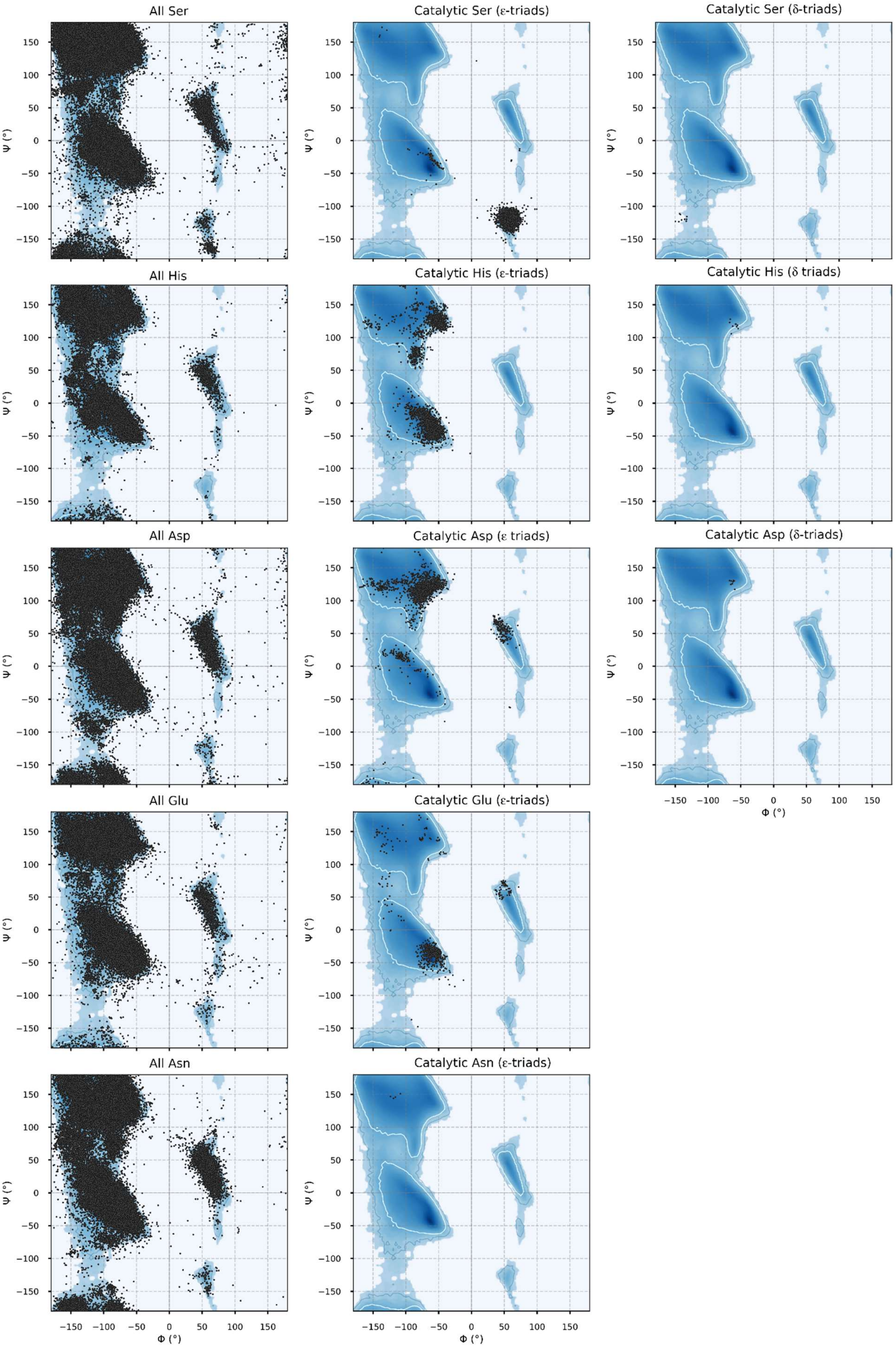

**Fig. S15: Ramachandran plots of control and active site residues in cysteine esterases / α/β-hydrolases.**

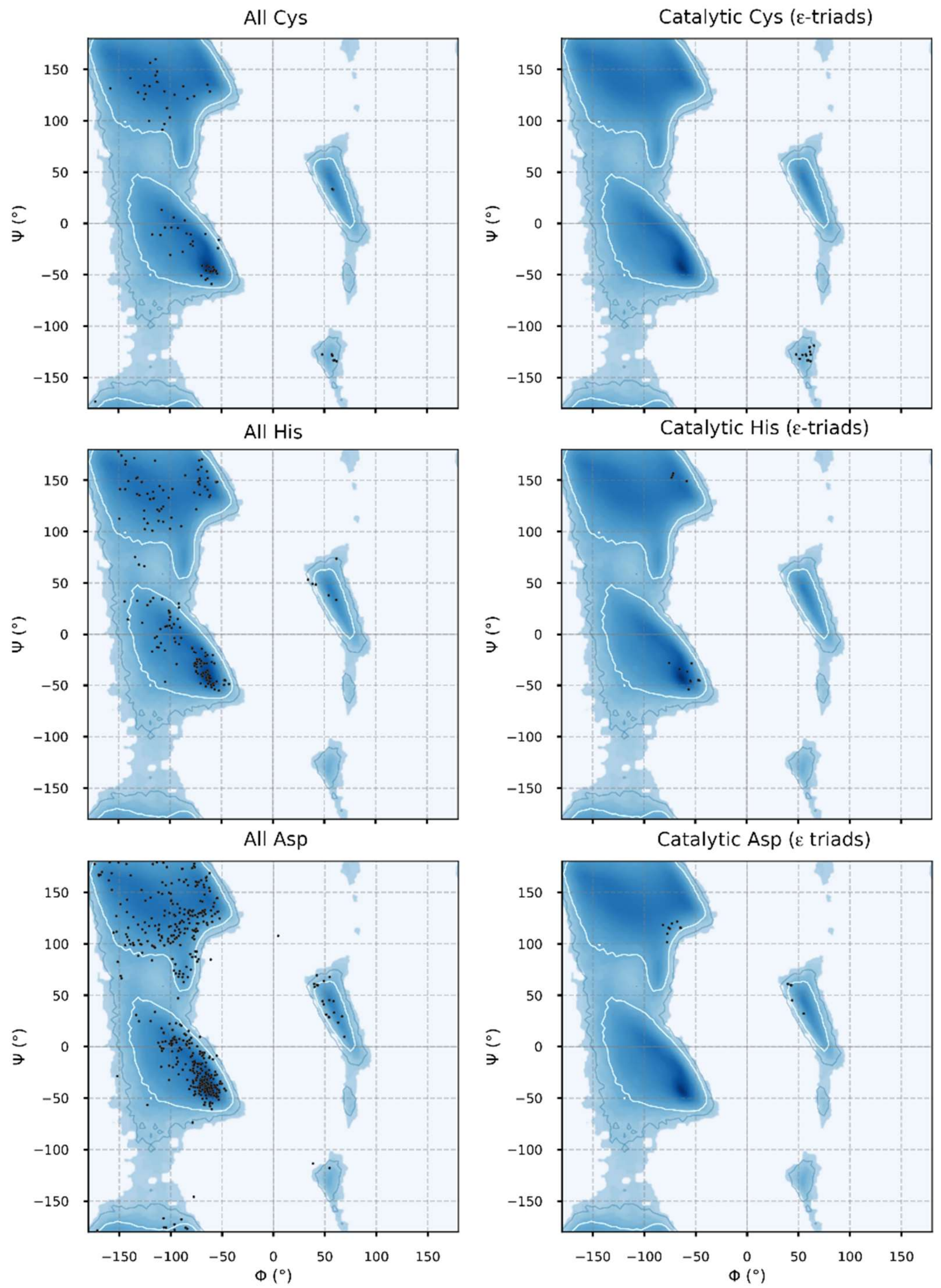

**Fig. S16: Ramachandran plot summary for control and active site residues in peptidases and esterases / α/β-hydrolases.** The Ramachandran plot assignments were collected from the validation.xml files downloaded from the PDB for the structures containing catalytic triads. The number of **(A) residues in disallowed regions** and **(B) residues in allowed (but not favored) regions** was divided by the total number of residues in the presented groups (in disallowed, allowed and favored regions). The high number of catalytic Ser/Cys residues in disallowed/allowed (but not favored) regions observed for peptidases and esterases / α/β-hydrolases results from the presence of some of these residues in the kink of the nucleophilic elbow. This feature is characteristic for α/β-hydrolase fold enzymes and corresponds to the type II′ β-turn additionally allowed region in the lower right quadrant of the Ramachandran plot.

A

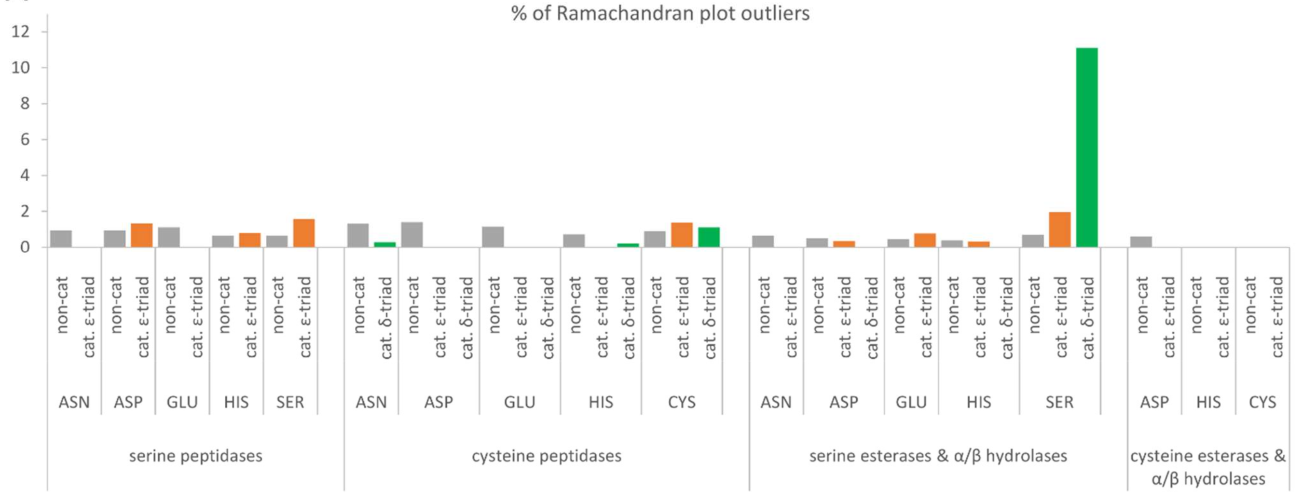


B

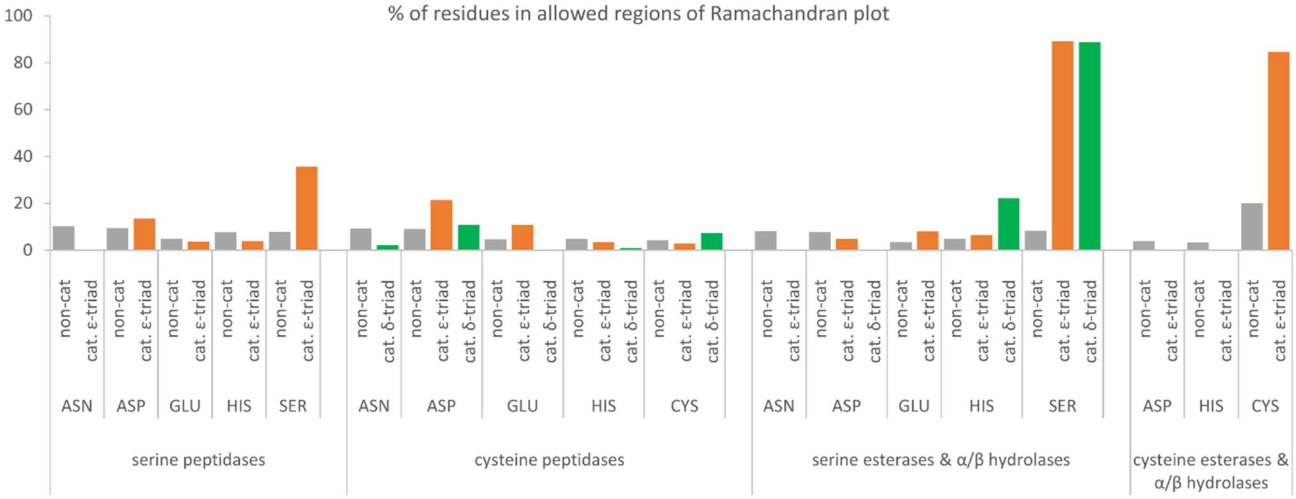

**Fig. S17: B-factor statistics of control and active site residues in peptidases and esterases / α/β-hydrolases.** The calculations were performed with the help of BAVERAGE program of the CCP4 suite[25]. The average B-factors of catalytic and non-catalytic residues were divided by the average B-factors of all non-heteroatoms in the structures containing the studied catalytic triads. The residues with B-factors below 1 Å$^2$ were eliminated as crystallographically implausible. Statistical analysis of the B-factor magnitudes would be invalid due to the confounding effect of radiation damage that depends on the side chain type and environment (disulfide bonding, etc.). However, the analysis of relative B-factors of histidines in serine and cysteine triads of matching type points to almost exactly the same values (differences < 1%), which suggests that more constrained conformations of serine catalytic triads are unlikely to be reflected in higher crystallographic temperature factors.

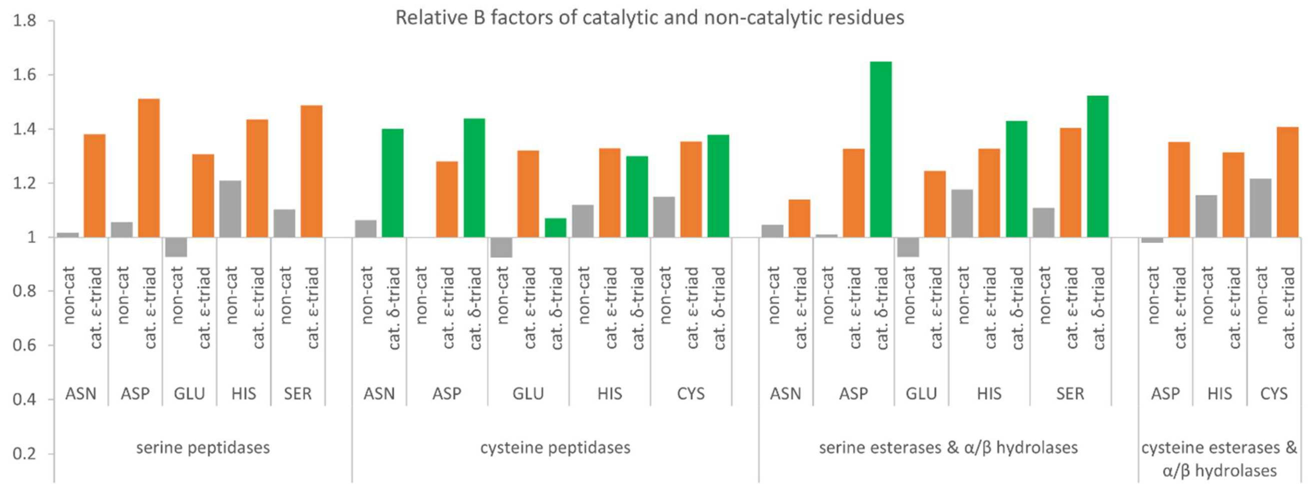

**Fig. S18: Triad configuration reversal models.** The models in **(A)** were obtained by a 180° flip of the histidine imidazole (the positions of the two nitrogen atoms were swapped). For the models in **(B)** an additional regularization of 10-30 residue long enzyme fragments containing the flipped histidine (with the positions of the histidine imidazole nitrogens fixed) was performed in the program COOT[23]. The nucleophile mutations in PDB entries 1lvb, 2zzp and 2io1 were reversed. The flipped histidine in MccF (PDB 3tlb) could not be easily connected with the rest of the protein. The 2.0 and 1.4 Å radii were assumed for CH2 and carbonyl O in agreement with the work of Richards[24]. Clashes with the enzyme scaffolds heavily depend on the extent of the regularization are thus not shown.

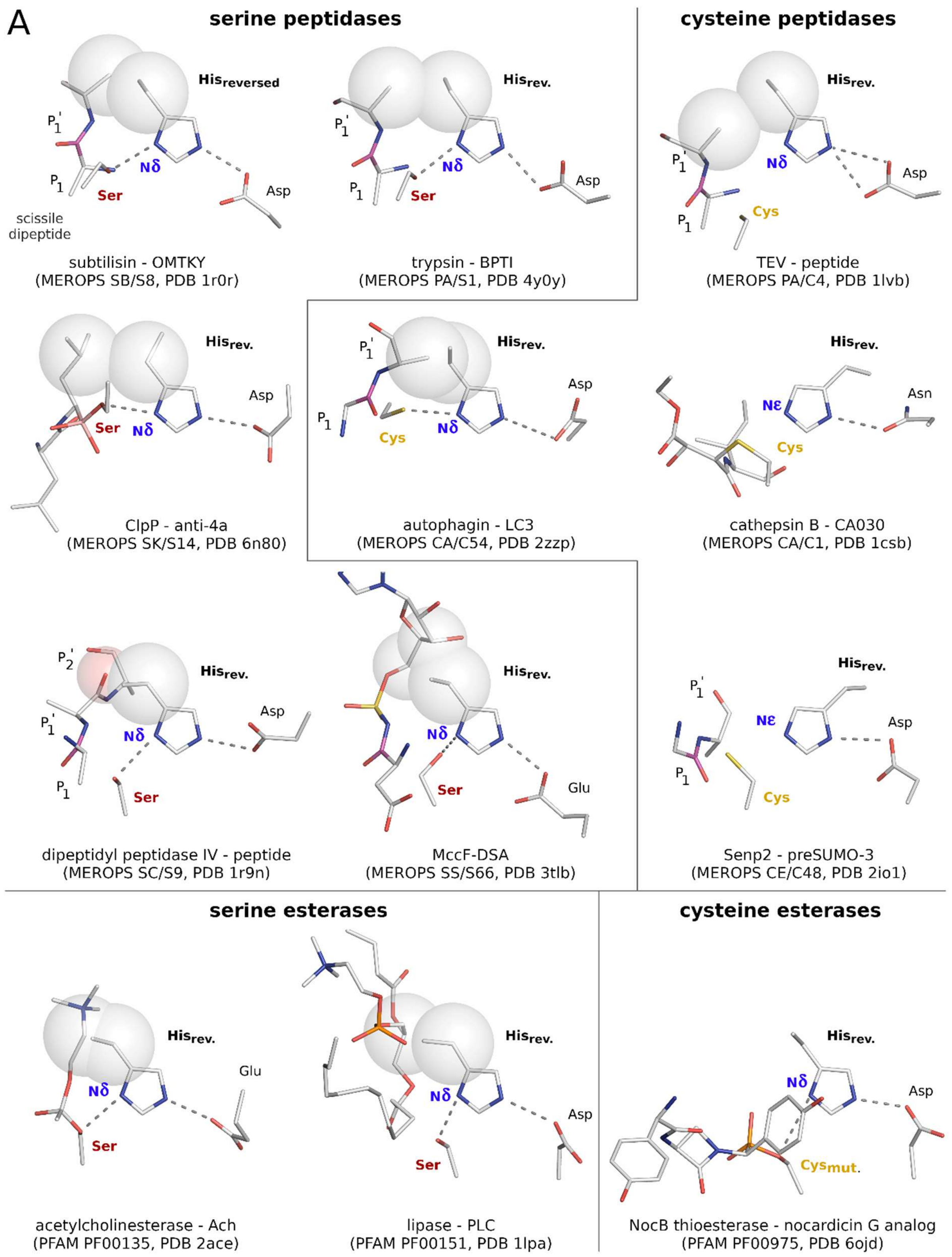

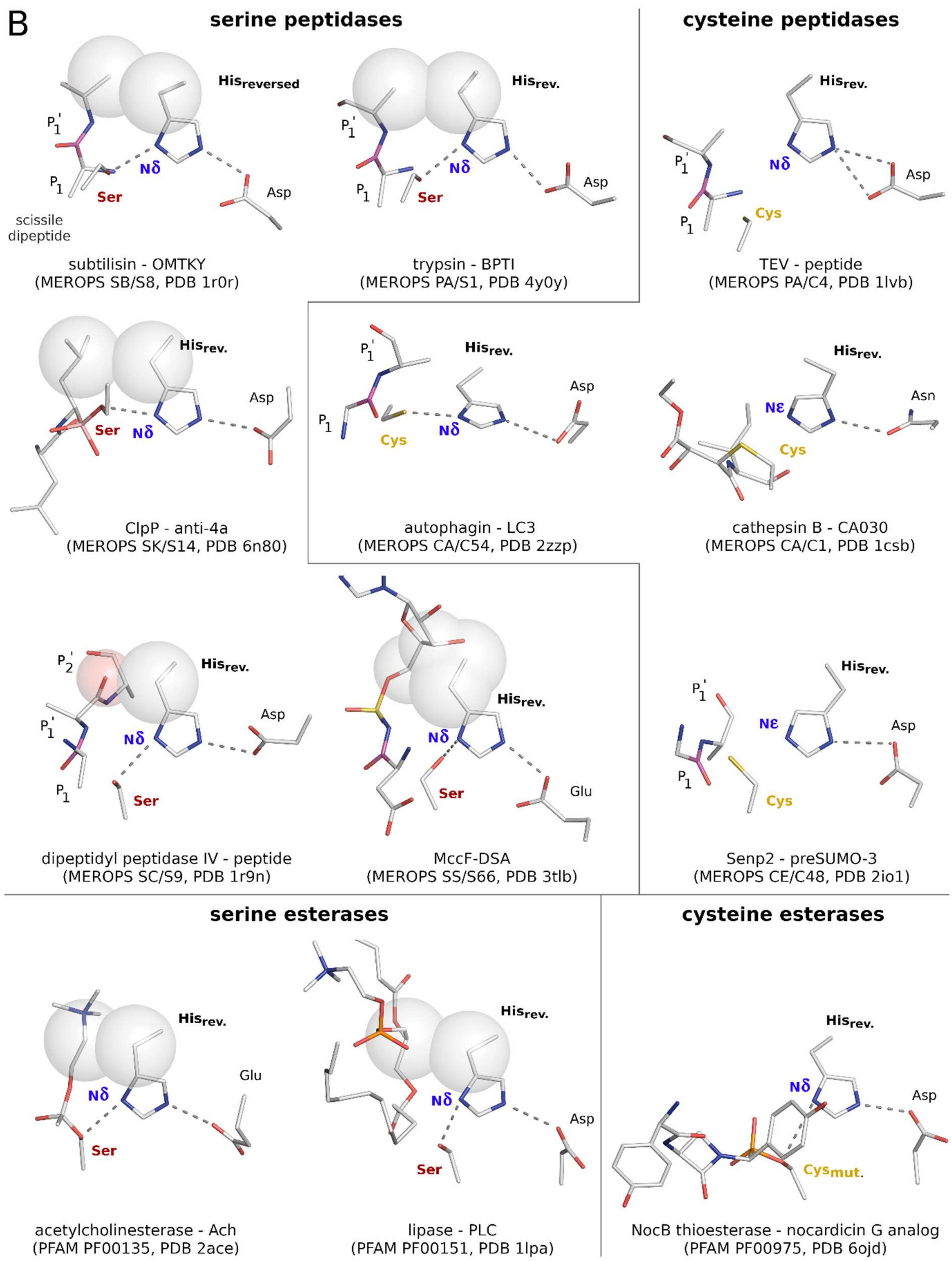
B
serine peptidases
cysteine peptidases
His reversed
P1'
P1
Nδ
Ser
Asp
scissile dipeptide
subtilisin - OMTKY
(MEROPS SB/S8, PDB 1r0r)
His rev.
trypsin - BPTI
(MEROPS PA/S1, PDB 4y0y)
Cys
TEV - peptide
(MEROPS PA/C4, PDB 1lvb)
ClpP - anti-4a
(MEROPS SK/S14, PDB 6n80)
autophagin - LC3
(MEROPS CA/C54, PDB 2zzp)
Nε
Asn
cathepsin B - CA030
(MEROPS CA/C1, PDB 1csb)
P2'
dipeptidyl peptidase IV - peptide
(MEROPS SC/S9, PDB 1r9n)
Glu
MccF-DSA
(MEROPS SS/S66, PDB 3tlb)
Senp2 - preSUMO-3
(MEROPS CE/C48, PDB 2io1)
serine esterases
cysteine esterases
acetylcholinesterase - Ach
(PFAM PF00135, PDB 2ace)
lipase - PLC
(PFAM PF00151, PDB 1lpa)
Cys mut.
NocB thioesterase - nocardicin G analog
(PFAM PF00975, PDB 6ojd)

**Fig. S19: The identity of the third triad residue in Nε- and Nδ-triads of (A) serine and (B) cysteine peptidases**. The cases were <u>weighted</u> based on sequence identity. The summary of the underlying data is presented in Tab. S7.

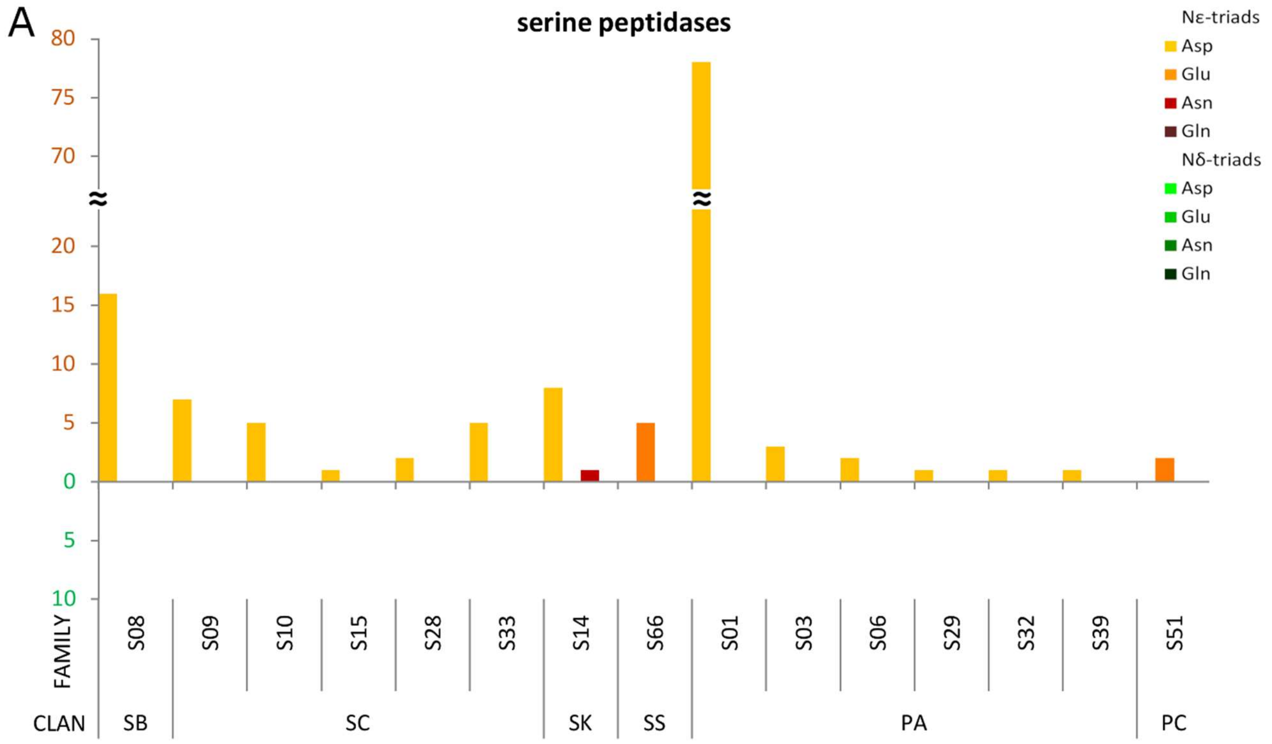


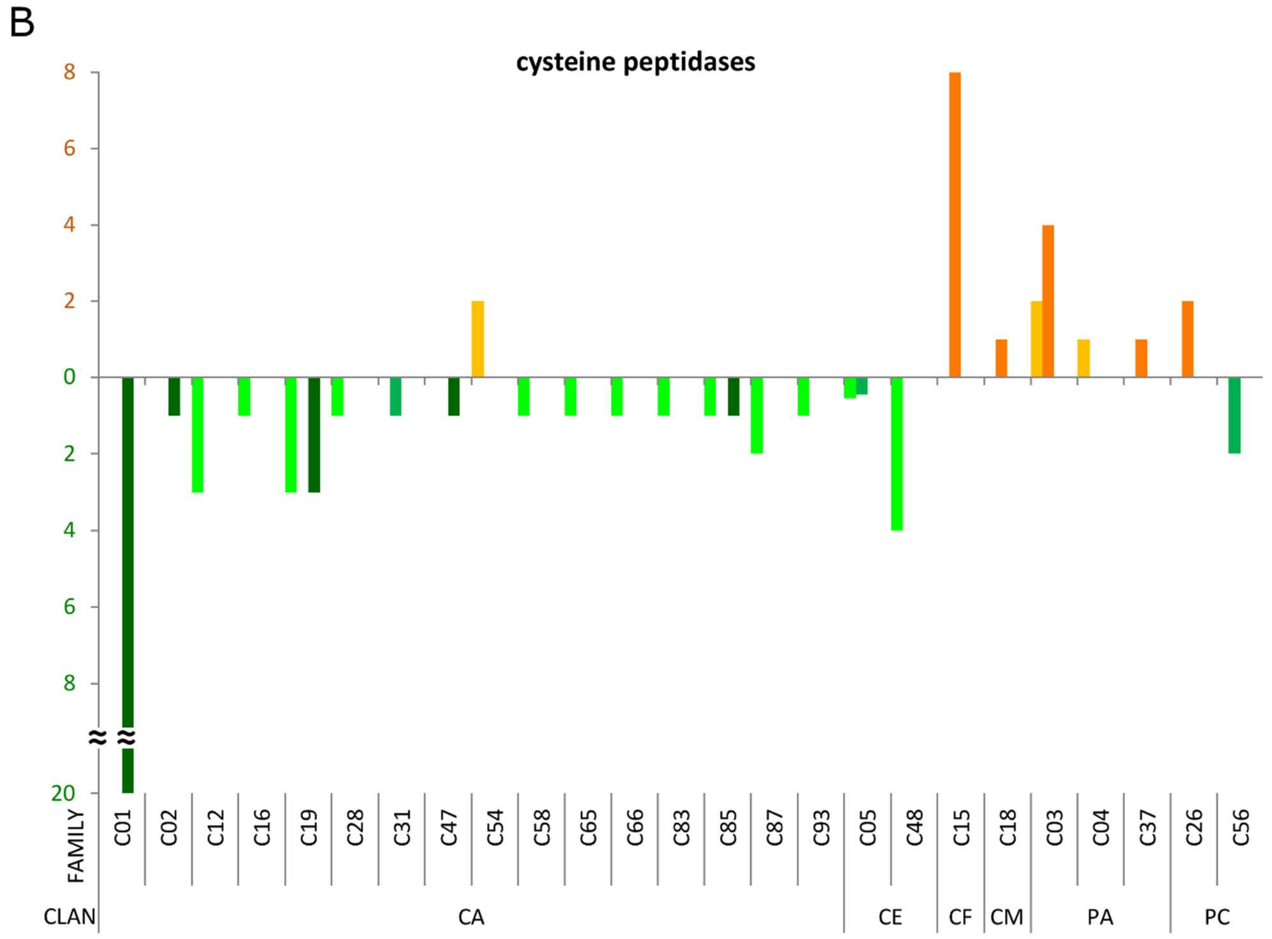

**Fig. S20: The identity of the third triad residue in Nε- and Nδ-triads of (A, B) serine and (C) cysteine esterases / α/β hydrolases**. The cases were weighted based on sequence identity. The summary of the underlying data is presented in Tab. S7.

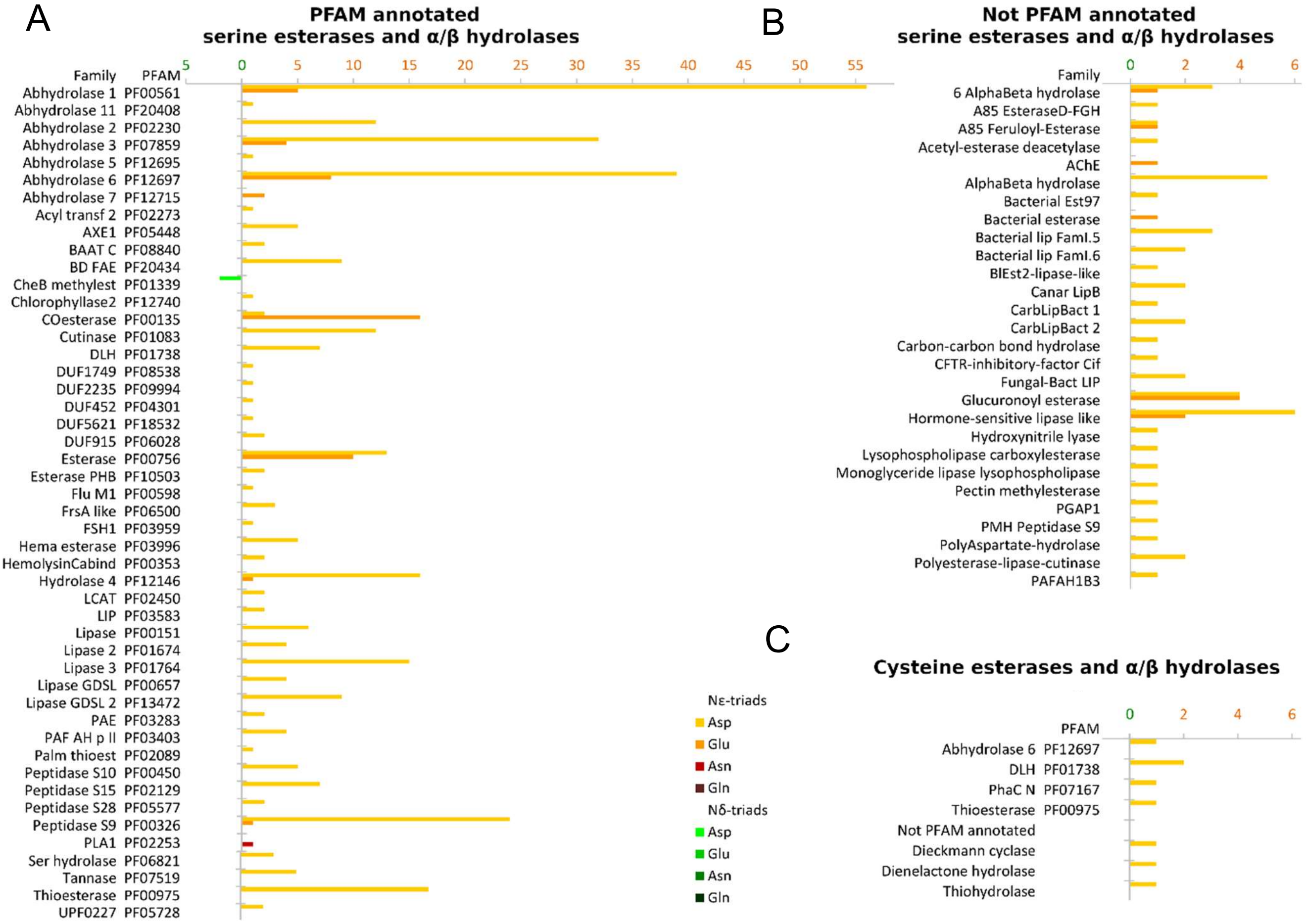


# Supplementary Tables

**Table S1. Statistics of putative triads found in the PDB (after the exclusion of peptidases and esterases).**

**(A) unweighted statistics**

| 1st triad residue | Nε-triads | Nδ-triads | Nδ-triads/Nε-triads | Unclear |
|---|---|---|---|---|
| Ser | 18409 | 27569 | 1.497 | 262 |
| Cys | 3796 | 4641 | 1.222 | 92 |
| Thr | 12550 | 22843 | 1.820 | 180 |
| All | 32657 | 53212 | 1.629 | 307 |

**(B) statistics weighted according to the CD-HIT clustering**

| 1st triad residue | Nε-triads | Nδ-triads | Nδ-triads/Nε-triads | Unclear |
|---|---|---|---|---|
| Ser | 1879.1 | 3282.5 | 1.746 | 21.2 |
| Cys | 435.8 | 576.9 | 1.323 | 7.0 |
| Thr | 1421 | 2701 | 1.900 | 12.9 |
| All | 3564.9 | 6324.7 | 1.774 | 25.4 |

## Table S2. Nδ-configured and unclear triads in serine peptidases

| MEROPS family | PDB code | Enzyme | Explanation for the triad arrangement | Remarks |
|---|---|---|---|---|
| S01.151 | 2j9n | trypsin | His incorrectly modelled in the density[M] | |
| S01.198 | 4kkd | MASP-3 | inactive catalytic center conformation | ~6 Å His-Ser distance |
| S01.216 | 1kig | factor Xa | His incorrectly modelled in the density[M] | |
| S01.216 | 2bq6 | factor Xa | His incorrectly modelled in the density[M] | |
| S01.216 | 2bq7 | factor Xa | His incorrectly modelled in the density[M] | |
| S01.216 | 4bti | factor Xa | His incorrectly modelled in the density[M] | |
| S01.217 | 1nro | α-thrombin | automatic classification confused by the presence of a receptor based peptide | Nε-triad (3.6 Å Nε-Ser and 3.3 Å Nδ-Asp distance) |
| S01.231 | 2vnt | UPA | unusual conformation of His in 3 out of 6 molecules in the asymmetric unit of the crystal | 4.5-5 Å His-Ser and His-Asp distances |
| S01.231 | 5wxf | UPA | automatic classification confused by the presence of an inhibitor | Nε-triad (2.8 Å Nε-Ser and 2.4 Å Nδ-Asp distance) |
| S01.233 | 1qrz | plasminogen | unusual His conformation in contact with non-catalytic Glu (proenzyme) | 3.5 - 4.2 Å His-Ser distance |
| S01.233 | 4a5t | plasminogen | 3.5 Å resolution was not good enough to correctly model the His conformation[M] | |
| S01.268 | 1p11 | alpha-lytic protease | unusual His orientation due to a covalently bound inhibitor | 5 Å His-Asp distance |
| S01.268 | 1p12 | alpha-lytic protease | unusual His orientation due to a covalently bound inhibitor | 6 Å His-Asp distance |
| S01.268 | 3qgj | alpha-lytic protease | unusual His orientation due to a covalently bound inhibitor | 7 Å His-Asp distance |
| S01.278 | 5m3o | mitochondrial serine protease HtrA2 | inactive catalytic center conformation | ~4 Å His-Asp distance (correct Nε triad in 5wyn structure) |
| S01.278 | 5tny | mitochondrial serine protease HtrA2 | inactive catalytic center conformation | |
| S01.278 | 5tnz | mitochondrial serine protease HtrA2 | inactive catalytic center conformation | |
| S01.278 | 5to0 | mitochondrial serine protease HtrA2 | inactive catalytic center conformation | |
| S01.438 | 1eq9 | chymotrypsin | unusual His orientation due to a covalently bound inhibitor | 5-6 Å His-Asp distance |
| S08.039 | 3gcx | PCSK9 | automatic classification confused by the presence of an inhibitory prodomain | Nε-triad (3.5 Å Nε-Ser and 2.9 Å Nδ-Asp distance) |
| S08.054 | 1ptk | proteinase K | $Hg^{2+}$ ion in the active site | |
| S08.054 | 3dvs | proteinase K | His incorrectly modelled in the density[A] | |
| S08.054 | 3dw1 | proteinase K | His incorrectly modelled in the density[A] | |
| S08.054 | 3dw3 | proteinase K | His incorrectly modelled in the density[A] | |
| S08.054 | 3dwe | proteinase K | His incorrectly modelled in the density[A] | |
| S08.054 | 3i2y | proteinase K | His incorrectly modelled in the density[A] | |
| S08.129 | 2z2y | subtilisin | $Zn^{2+}$ ion in the active site | Ser to Cys mutation |
| S08.129 | 3a3n | subtilisin | $Zn^{2+}$ ion in the active site | Ser to Ala mutation |
| S09.003 | 5y7h | DPP4 | His incorrectly modelled in molecule B[A] | |
| S14.001 | 3tt6 | ClpP | inactive catalytic center conformation | ~7 Å His-Ser distance |
| S14.001 | 4jcq | ClpP1 | inactive catalytic center conformation | >4 Å His-Ser distance |
| S14.001 | 4ryf | ClpP1/2 | His incorrectly modelled in the density[R] | |
| S14.001 | 5dkp | ClpP | His inconsistently modelled in the density[*] | |
| S14.011 | 2f6i | ClpP | inactive catalytic center conformation | >7 Å His-Ser distance |
| S29.001 | 4b6f | HCV NS3 | His incorrectly modelled in the density[M] | |
| S29.001 | 4b76 | HCV NS3 | His incorrectly modelled in the density[M] | |

[M]According to MolProbity[26] validation [A]According to Auto Fit Rotamer in COOT[23] [R]His Cβ flip necessary and real space refinement in COOT[23] [*]Diffraction data not good enough to cleanly model the His side chain despite 2.38 Å resolution (His in some subunits correctly modelled, in some should be flipped according to MolProbity, in some should be flipped according to PDB-REDO[27], in some has inactive conformation but the active one fits the density).

**Table S3. Enzymes other than peptidases containing putative catalytic triads found with the help of M-CSA[28] and EzCatDB[29] databases.**

### (A) Serine triad containing enzymes

| EC number | M-CSA/EzCatDB[a] | Enzyme example | Triad/Dyad | EC class |
|---|---|---|---|---|
| | | **Nε-configured triads** | | |
| 1.11.1.10 | 248 | chloride peroxidase (co-factor free family) | SHD | oxidoreductase |
| 2.3.1.39 | 291 | malonyl-CoA-acyl carrier protein transacylase | SH | transferase |
| 2.3.1.94 | 602 | 6-deoxyerythronolide-B synthase | SHD | |
| 2.3.1.- | 728 | myristoyl-ACP-specific thioesterase | SHD | |
| 3.1.1.- | 900 | para-nitrobenzyl esterase | SHE | hydrolase |
| 3.1.1.1 | 407 | carboxylesterase | SHD | |
| 3.1.1.2 | S00720 | aryl-ester hydrolase PFE | SHD | |
| 3.1.1.3 | 218,518,519,520 | triacylglycerol lipase | SHD/E | |
| 3.1.1.4[b] | 650 | phospholipase A1 | SHN | |
| 3.1.1.5[b] | 755 | lysophospholipase | SHD | |
| 3.1.1.7 | 922 | acetylcholinesterase | SHE | |
| 3.1.1.13[b] | 401 | bile salt-activated lipase | SHD | |
| 3.1.1.41[b] | 866 | cephalosporin-C deacetylase | SHD | |
| 3.1.1.43 | 666 | alpha-amino-acid esterase | SHD | |
| 3.1.1.47 | 94 | 1-alkyl-2-acetylglycerophosphocholine esterase | SHD | |
| 3.1.1.72 | 431 | acetylxylan esterase | SHD | |
| 3.1.1.73 | S00723 | cinnamoyl esterase | SHD | |
| 3.1.1.74 | 631 | cutinase | SHD | |
| 3.1.1.84 | 465 | cocaine esterase | SHD | |
| 3.1.1.86 | 705 | rhamnogalacturonan acetylesterase | SHD | |
| 3.1.2.14 | - | thioesterase domain of human fatty acid synthase | SHD | |
| 3.1.2.- | S00919 | thioesterase | SHD | |
| 3.1.2.22 | 522 | palmitoyl[protein] hydrolase (type 1) | SHD | |
| 3.1.2.- | 523[c] | palmitoyl[protein] hydrolase (type 2) | SHD | |
| 3.7.1.8 | 437 | 2,6-dioxo-6-phenylhexa-3-enoate hydrolase | SHD | |
| 3.7.1.9 | 771[d] | 2-hydroxymuconate-semialdehyde hydrolase | SHD | |
| 4.1.2.47 | 217 | (S)-hydroxynitrile lyase | SHD | lyase |
| 4.2.99.20 | - | SHCHC synthase | SHD | |
| | | **Nδ-configured triads** | | |
| 2.8.1.2 | 871 | 3-mercaptopyruvate sulfurtransferase | SHD[e] | transferase |
| 3.1.1.61[b] | 337 | protein-glutamate methylesterase (CheB) | SHD | hydrolase |
| 5.4.2.2 | 352 | phosphoglucomutase | SH | isomerase |
| 5.4.2.8[b] | 194 | phosphomannomutase/phosphoglucomutase | SH | |

[a] EzCatDB numbers are provided if the M-CSA entry was missing. [b] Additional EC numbers listed in M-CSA. [c] The example structure in M-CSA has wrong His side chain rotamer. [d] The example structure in M-CSA depicts the active site S->A mutant. [e] Both Nδ- and Nε-configuration has been modelled. Cysteine is the nucleophile and SHD triad only activates it.

## (B) Cysteine triads

| EC number | M-CSA/EzCatDB | Enzyme example | Triad/Dyad | EC class |
|---|---|---|---|---|
| **Nε-configured triads** | | | | |
| 1.11.1.1 | 895 | NADH peroxidase | CH | oxidoreductase |
| 1.2.1.11 | 429 | aspartate-semialdehyde dehydrogenase | CH | |
| 1.2.1.12 | 911 | glyceraldehyde-3-phosphate dehydrogenase (phosphorylating) (type I) | CH | |
| 1.2.1.59 | 619 | glyceraldehyde-3-phosphate dehydrogenase (NAD(P)+) (phosphorylating) | CH | |
| 1.2.1.41 | D00614[a] | gamma-glutamyl phosphate reductase | CHE | |
| 1.4.4.2[b] | M00163 | glycine dehydrogenase (decarboxylating) | CHE | |
| 1.8.1.4 | T00017 | dihydrolipoyl dehydrogenase | CHE | |
| 1.8.1.7 | 6 | glutathione-disulfide reductase | CHE[c] | |
| 1.8.1.12 | T00242 | trypanothione reductase (TR) | CHE | |
| 2.3.1 | - | polyhydroxyalkanoate (PHA) synthase PhaC | CHE | transferase |
| 2.3.1.16 | 77 | acetyl-CoA C-acyltransferase | CH | |
| 2.3.1.74 | 355 | chalcone synthase | CH | |
| 2.3.1.179 | 574 | beta-ketoacyl-acyl-carrier-protein synthase II | CH | |
| 2.8.1.7[b] | 855 | cysteine desulfurase | CH | |
| 3.1.1.45 | 492 | carboxymethylenebutenolidase | CHD | hydrolase |
| 4.1.3.27 | 314 | Anthranilate synthase | CHE | |
| 4.2.3.12 | 84 | 6-pyruvoyltetrahydropterin synthase | CHE[c] | lyase |
| 6.3.5.2 | 234 | GMP synthase (glutamine-hydrolysing) | CHE | |
| 6.3.5.13[b] | - | glutamine amidotransferase GatD | CHE | ligase |
| **Nδ-configured triads** | | | | |
| 2.1.1.63 | 251 | O6-alkylguanine-DNA alkyltransferase | C(HOH)HE | transferase |
| 2.3.1.5[b] | 515 | N-hydroxyarylamine O-acetyltransferase | CHD | |
| 2.3.2.13 | 149 | protein-glutamine gamma-glutamyltransferase | CHD | |

[a] The structures in EzCatDB are not representative. [b] Additional EC numbers listed. [c] Triad composed of two polypeptide chains.

## Table S4. *In vitro* attempts of Ser/Cys nucleophile switch.

## (A) Ser->Cys engineering

| Enzyme | MEROPS clan | MEROPS fam. | Triad | Triad type | Mut. | Activity loss/ gain | Ref. | Catalytic effect |
|---|---|---|---|---|---|---|---|---|
| **peptidases** | | | | | | | | |
| trypsin | PA | S01 | SHD | Nε | S195C | -- | [30] | Trypsin S195C shows a low level of activity toward the activated ester substrate Z-Lys-pNP, while both trypsin S195C and trypsin D102N,S195C were active toward the fluorogenic tripeptide substrate Z-GPR-AMC.<br>The kcat of trypsin S195C was reduced by a factor of 6.4 x 10^5 relative to that of trypsin while the kcat of trypsin D102N,S195C was lowered by a factor of 3.4 X 10^7 with Z-GPR-AMC as substrate. Km values were unaffected. |
| | | | | | | | [31] | **Using the sensitive fluorogenic substrate, Z-GPR-AMCT, the kcat of the reduced form of trypsin-S195C was found to be 6.4 x 10^5 lower than that of trypsin (Higaki et al, 1989), but still about four orders of magnitude above aqueous hydrolysis** (Kahne & Still. 1988). |
| *S. griseus* trypsin | PA | S01 | SHD | Nε | S195C | -- | [32] | It exhibited **only traces of catalytic activity toward typical trypsin substrates such as Nalpha-tosyl-L-arginine methyl ester, whereas it retained some activity toward "active ester" substrates** such as Nalpha-carbobenzoxy-L-lysine p-nitrophenyl ester. |
| exfoliatin A | PA | S01 | SHD | Nε | S195C | -- | [33] | To demonstrate that this serine is critical in the cleavage of hDsg1, it was mutated to a cysteine (resultant mutant ETA Cmu). Incubation of ETA Cmu with Dsg1 at 37°C for 1 hour demonstrated a markedly decreased rate of cleavage compared with wild-type ETA |
| togavirin | PA | S03 | SHD | Nε (dist.) | S215C | - | [34] | Substitution with Cys resulted in 60% cleavage, and it is probable that the -SH of Cys functions in the same fashion as the -OH of Ser in this protease. |
| flavivirin | PA | S07 | SHD | Nε | S138C | -- | [35] | Two mutations that either abolish (Ser-138 to Ala) or significantly reduce (Ser-138 to Cys) the NS2B3.1 cleavage activity in vitro were analyzed in vivo by transfection of BHK cells with RNA transcripts derived from full-length YF cDNA templates containing these mutations |
| subtilisin | SB | S08 | SHD | Nε | S221C | --- | [36] | The hydroxyl of the serine in the active center of subtilisin has been converted to a sulfhydryl group. The resulting enzyme, thiol-subtilisin, is at most 1/100 as active as native subtilisin toward normal ester and peptide substrates.<br>The conversion of the serine residue at the active site of subtilisin to cysteine produces a protein which still reacts with nitrophenyl acetate but fails to react with any of the classical substrates of the enzyme, including proteins. |
| eryngase | SC | S09 | SHD | Nε (pred.) | S524C | --- | [37] | In contrast to wild type, S524C mutant enzyme exhibited no hydrolysis reaction |
| S9AP aminopeptidase | SC | S09 | SHD | Nε (pred.) | S502C | --- | [38] | The site-directed mutagenesis of catalytic Ser502 into Cys caused the disappearance of its aminopeptidase activity |
| peptidase E | PC | S51 | SHD | Nε | S120C | -- | [39] | Conversion of Ser120 to Cys leads to severely reduced (10^4-fold) but still detectable activity |
| **esterases & other enzymes** | | | | | | | | |
| acetylcholine-esterase | | | SHE | Nε | S200C | -- | [40] | Mutation of serine-200 to cysteine results in diminished activity |
| | | | | | | | [41] | Engineering of triads such as Cys-His-Glu or Ser-His-Asp resulted in reduced productivity and in non-detectable (less than 1% of wild type) cholinesterase activity. Substitution of active-site Ser by Cys in TcAChE (Gibney et al., 1990) was reported previously to yield AChE with detectable activity, yet the catalytic efficiency of the mutant was approx. 100-fold lower than that of wild type. In contrast, replacement by Ser of the Cys residue which serves as nucleophile in the dienelactone hydrolase triad (Pathak and Ollis, 1990) did not lead to activity loss beyond 10% of wild type (Pathak et al., 1991). This could indicate a shift toward a catalytic mechanism of higher stringency during evolution of the cholinesterases, which is not |

| | | | | | | | | |
|---|---|---|---|---|---|---|---|---|
| | | | | | | | | observed in the dienelactone hydrolase of Pseudomonas. |
| cocaine esterase | | | SHD | Nε | S117C | --- | [42] | S117C mutation, which results in both a drastically lowered kcat and increased KM |
| LuxD thioesterase | | | SHD | Nε | S114C | --- | [43] | The conversion of the active site serine of LuxD into cysteine (S114C) decreased the turnover of the enzyme with p-nitrophenyl myristate and myristoyl-CoA to close to background levels. |
| thioesterase domain of chicken fatty acid synthase | | | SHD | Nε | S101C | - | [44] | the Ser101->Cys mutant enzyme (thiol-thioesterase) retained considerable activity |
| thioesterase II | | | SHD | Nε | S101C | - | [45]<br><br>[46] | Replacing Ser101 with Cys yielded a mutant enzyme (thiol-TEII) that retained considerable catalytic activity and displayed properties of a cysteine thioesterase similar to those obtained with thiol-TE1 (3).<br><br>The S101C thioesterase II mutant had significant activity with either model, acyl-CoA and acyl-p-nitrophenol, or natural, acyl-(fatty acid synthase), substrates (Table I). Values of kcat were 20-90% of those of the wild-type enzyme; however, the Km values were reduced so that the values for the specificity constants, kcat/Km, decreased only about 2-fold for acyl-(fatty acid synthase) and actually increased by almost 2-fold for decanoyl-CoA. In the case of p-nitrophenyldecanoate, the kinetic constants were very similar for the wild-type and mutant enzymes. |
| pikromycin thioesterase | | | SHD | Nε | S148C | + | [47] | Kinetic analysis revealed that the serine to cysteine substitution afforded a superior cyclization catalyst for each substrate tested. Pik TES148C displayed a 4.3- and 12-fold increase in the kcat/Km for macrolactonization of both the native (3) and C-3 methoxy hexaketide 4 compared to Pik TEWT, respectively. In reactions containing methyl-protected epimerized 5, not only did Pik TES148C retain the ability to catalyze macrolactonization of the linear substrate, but notably the kcat/Km was 5.5-fold higher than for Pik TEWT-catalyzed hydrolysis. |
| cytosolic type I acyl-CoA thioesterase | | | SHD | Nε | S232C | -- | [48] | Interestingly, the S232C CTE-I mutant retained about 2% of the wild type activity, which was still about 200-fold higher than the activity of the other mutants. The acylation experiments showed that wild-type CTE-I is not acylated under the conditions used, whereas the S232C mutant becomes very strongly acylated. In addition, the S232A mutant was not acylated |
| hormone-sensitive lipase | | | SHD | Nε | S423C | --- | [49] | the Ser-423 mutants were devoid of both lipase and esterase activity |
| *Staphylococcus hyicus* lipase | | | SHD | Nε (pred.) | S369C | --- | [50] | Substitution by site-directed mutagenesis of this serine (Ser369) by a cysteine resulted in a mutant with only 0.2% residual activity. |
| *Candida antarctica* lipase B | | | SHD | Nε | S105C | -<br>++ with add. mut. | [51] | Kinetic experiments using purified variant S105C (QW1) show that this mutation diminishes the catalytic efficiency by a factor of about two, with $k_{cat}/K_m$ (WT CALB) being 150 $s^{-1}M^{-1}$ compared to $k_{cat}/K_m = 88\ s^{-1}M^{-1}$ for mutant QW1<br>An extremely high catalytic efficiency was observed for the purified variant QW4 (W104V/S105C/A281Y/A282Y/V149G) ($k_{cat}/K_m = 5920\ s^{-1}M^{-1}$), with $k_{cat}$ increased by 6 folds and catalytic efficiency ($k_{cat}/K_m$) increased by 40 folds compared with WT |
| lipoprotein lipase | | | SHD | Nε | S132C | --- | [52] | The native enzyme and mutant LPL in which serine 132 was replaced with alanine, cysteine, or glycine were transiently expressed in COS-7 cells. Mutant proteins were synthesized and secreted at levels comparable to native LPL; however, none of the mutants retained enzymatic activity |
| BphD C-C bond hydrolase | | | SHD | Nε | S112C | -- | [53] | estimates of initial velocities at 25 °C generate kcat = 0.27 +/- 0.02 s-1 and Km = 2.8 +/- 0.6 uM and a specificity constant kcat/Km = 9.5 +/- 2.2 × 10^4 M-1 s-1, approximately 240-fold lower than that of the wild-type enzyme. |
| *E. coli* outer membrane phospholipase A | | | SHN | Nε | S144C | -- | [54] | Replacement of Ser144 by cysteine resulted in 1% residual activity, whereas the other substitutions at this position yielded virtually inactive enzymes. |

## (B) Cys->Ser engineering

| Enzyme | MEROPS | | Triad | Triad type | Mut. | Activity loss/ gain | Ref. | Catalytic effect |
|---|---|---|---|---|---|---|---|---|
| | clan | fam. | | | | | | |
| **peptidases** | | | | | | | | |
| papain | CA | C01 | CHN | Nδ | C25S | --- | [55]<br><br><br>[56] | Ser25-papain and dehydro-Ser25-papain show no enzymatic activity towards N-benzyloxycarbonyl-glycine P-nitrophenyl ester or N-benzyloxycarbonyl-L-lysine p- nitrophenyl ester which are good substrates for the native enzyme.<br>active site cysteine to serine mutation eliminated the proenzyme's ability to autoactivate.<br><br>[Ser25]Papain and [dSerZ5]papain show no enzymic activity towards N-benzyloxycarbonylglycine p-nitrophenol ester or N-benzyloxycarbonyl-L-lysine p-nitrophenyl ester which are good substrates for papain [18,31]. |
| cathepsin K | CA | C01 | CHN | Nδ | C139S (+S163 A) | --- | [57] | Procathepsin K (containing mutation C139S,S163A) failed to spontaneously process and was only partially processed in the presence of 1% exogenous wild-type mature cathepsin K |
| cathepsin B | CA | C01 | CHN | Nδ | C29S (+S115 A) | --- | [58] | This non-active mutant protein was secreted essentially in an unprocessed form.<br>No cathepsin B activity was detected in the culture medium. |
| cathepsin L | CA | C01 | CHN | Nδ | C25S | --- | [59] | Cys25Ser mutant was incapable of self-processing |
| | | | | | | | [60] | the mature form of the mutant was totally inactive against the quenched fluorogenic cathepsin S peptide substrate Abz-Leu-Thr-Bal-Hyp-Tyr($NO_2$)-Asp-$NH_2$. |
| *Sphenophorus levis* pseudo-cysteine protease | CA | C01 | CHN | nd | S138C | --- | [61] | The Sl-CathL-CS did not display any activity on complex, specific serine and cysteine protease fluorimetric substrates. The reversion of Ser138 to Cys138 in Sl-CathL-mutSC provided the protein with the capability of degrading skim milk powder and hydrolyzing Z-Phe-Arg-AMC with Km = 10.77 mM and Vmax = 1017.60 ± 135.55 (Figure 3C), but not Z-Leu-Arg-AMC and Z-Arg-Arg- AMC. |
| calpain | CA | C02 | CHN | Nδ | C105S | --- | [62]<br><br>[63] | Mutants Cys105Ser, His262Ala and Asn286Ala had no activity.<br><br>m-105S-calpain does not degrade casein nor an artificial tetra-peptide substrate, succinyl-Leu-Leu-Val-Tyr-MCA. Further, it shows no autolytic activity with Ca2+. |
| DEK1 phytocalpain | CA | C02 | CHN | Nδ (pred.) | C71S | --- | [64] | Activity measurements show that the mutant protein is inactive compared with the wild-type protein |
| CvaB | CA | C39 | CHD | Nδ (pred.) | C32S | --- | [65] | neither the serine nor alanine mutant had the colicin V secretion activity of the wild type |
| lantibiotic protease | CA | C39 | CHD | Nδ (pred.) | C12S | --- | [66] | Mutation of Cys12 to either Ser or Ala resulted in loss of all proteolytic activity |
| phytochelatin synthase | CA | C83 | CHD | Nδ | C56S | --- | [67]<br><br><br><br><br><br><br>[68] | None of the Cys-to-Ser (or -Ala) or Ser-to-Ala substitutions, except for the C56S and C56A substitutions, decreased the capacity of heterologously expressed AtPCS1-FLAG to confer Cd2 tolerance or to mediate PC synthesis<br>we found that Cys-to-Ala or -Ser mutagenesis of only one of these residues (Cys56) abolished the suppression of Cd2 hypersensitivity and Cd2-dependent intracellular PC accumulation otherwise conferred by the heterologous expression of wild-type AtPCS1-FLAG in S. cerevisiae. We have repeated our experiments many times, repeatedly double-checked the fidelity of the constructs used by direct sequencing, and consistently obtained the same results.<br><br>In marked contrast, C56S-mutated AtPCS1-FLAG confers a degree of Cd21 tolerance only marginally greater than that of empty- |

| | | | | | | | | |
|---|---|---|---|---|---|---|---|---|
| | | | | | | | | vector pYES3 controls, and cell-free extracts prepared from these transformants exhibit negligible PC synthetic activities |
| RTX self-cleaving toxin | CD | C80 | C(HOH) HD | Nδ | C3568S | --- | [69] | A cysteine point mutation within the RTX holotoxin attenuated actin crosslinking activity suggesting that processing of the toxin is an important step in toxin translocation. |
| MCF | CD | C80 | CHD | Nδ | C3351S (N-ter) | --- | [70] | When either of the catalytic residues was mutated, MCF completely lost autocleavage activity in HEK293T cells |
| *Pasteurella multocida* toxin putative cysteine peptidase | - | C116 | CHD | Nδ | C1165S | --- | [71] | The substitution of Cys1165 with serine, glycine, or arginine abolishes the PMT activities.<br>PMT toxicities on target cells were completely abrogated when one of the amino acids constituting the triad was mutated. |
| Cif type III effector | - | - | CHQ | Nδ | C156S/ C109S/ C128S | --- | [72] | Ectopic expression of the catalytic cysteine mutants (Cif C109S, CHBP C156S, or CHPL C128S) in 293T cells failed to induce a G2/M arrest in contrast to their wild-type counterparts (Fig. 5A). Next, when equal amounts of recombinant wild-type CHBP or the catalytic triad mutant (C156S, H211N, or Q231A) were delivered directly into HeLa cells synchronized at the G1/S boundary, all of the catalytic triad mutants appeared to lose the ability to inhibit the cell cycle progression (Fig. 5B). Finally, human EPEC strain E2348/69 harboring wild-type Cif induced an evident G2/M arrest. In contrast, mutations of the catalytic triad of Cif (C109S, C109A, H165N, and Q185A) abolished its cell cycle arrest activity during EPEC infection |
| poliovirus-type picornain 3C | PA | C03 | CHE | Nε | C147S | -- | [73]<br>[74]<br>[75]<br>[76]<br>[77] | Mutations altering the strongly conserved residues (Cys-147 and His-161) exhibited a significant decrease in the proteolytic activity as assayed in the E. coli system.<br>The activity of mutated 3C proteases was in the following hierarchy: Glu71-Cys147 (wild type) > Asp71-Cys147 > Glu71-Ser147 > Gln71-Cys147 > Asp71-Ser147 > Gln71-Ser147 (inactive at all sites).<br>Single substitutions of the residues His-40, Glu-71, and Cys-147 by Tyr, Gln, and Ser, respectively, resulted in an inactive enzyme.<br>Transformation of poliovirus 3C peptidase into a serine peptidase results in lower activity by a factor of 430, but the activity extends toward higher pH with the more basic hydroxyl group.<br>C147S mutation does not abrogate 3C activity on nonstructural polypeptides<br>The relatively low rate of wild-type 3C-mediated processing that occurs after cycloheximide-mediated inhibition of translation suggests that nonstructural protein processing is carried out in a predominantly cis-like fashion, possibly in a complex formed by the nascent poly-protein. Alternatively, efficient processing of nonstructural proteins may be linked to active translation. |
| human rhinovirus protease 3C | PA | C03 | CHE | Nε | C146S | --- | [78]<br>[79] | if Cys-146 of HRV-14 3Cpro (equivalent to poliovirus Cys-147) was changed either to serine, methionine, or threonine, proteolytic activity was completely destroyed<br>When the mutant protease 3C (Cys146->Ser) was incubated with peptide II, no cleavage occurred. |
| grapevine fanleaf nepovirus picornain 3C | PA | C03 | CHE | Nε (pred.) | C179S | -/+ | [80] | replacement by a serine at the same position produced a mutant with an activity identical to that of native proteinase |
| 3C proteinase of coxsackievirus B3 | PA | C03 | CHE | Nε | C147S | - | [81] | The substitution of Cys147 with Ala abolished 3Cpro activity while the mutant in which Cys147 was replaced with Ser retained reduced proteolytic activity both in cis and in trans.<br>When the substrate peptide was incubated with a high concentration of the C147S mutant 3Cpro (a 15-fold higher concentration than that of wild type 3Cpro), greatly reduced but detectable amounts of the cleavage products were generated (Figure 4C, peaks 2 and 3). This result is the first demonstration that the C147S mutant of picornavirus 3Cpro can cleave a substrate in trans although the activity is greatly impaired in this mutant. One reason why other investigators failed to detect the peptidolytic activity of the C147S mutant 3Cpro may be that the enzyme concentrations they used (1-2 uM) were low (Knott et al., 1989; Hammerle et al., 1991). We detected negligible levels of cleavage products using similarly low concentrations of the C147S mutant 3Cpro. The activity of the C147S mutant 3Cpro was not inhibited by the presence of 5 mM iodoacetamide (Figure 4E) |

| | | | | | | | | |
|---|---|---|---|---|---|---|---|---|
| | | | | | | | | which almost completely inhibited wild type 3Cpro (Figure 4D). These results strongly suggest that the activity of the C147S mutant 3Cpro is not derived from any wild type enzyme which may have contaminated the mutant enzyme preparation<br>The substitution of Cysl47 with Ala abolished the autocatalytic activity of 3Cpro in E.coli. In contrast, the C147S mutant 3Cpro showed significant autocatalytic activity and very reduced, but detectable level of peptidolytic activity. |
| hepatitis A virus protease 3C | PA | C03 | CHD | Nε | C172S | --- | [82]<br><br><br><br>[83] | the standard proteolytic cascade operating on P3 polyprotein was completely inhibited by the H44Y, the C172G, and the C172S substitutions (Fig. 6). In each case, the variably programmed IVTTs produced P3 polyprotein effectively. However, all other peptides (i.e., 3ABC, 3BC, and 3C pro ) of the standard processing pattern were absent even after prolonged times of reaction (i.e., 12 h)<br><br>In both the peptide-based Hepatitis Virus Proteinase assays and the precursor-processing studies, the C172A mutant showed absolutely no proteolytic activity. |
| foot-and-mouth disease virus 3C proteinase | PA | C3 | CHD | Nε | C163S | - | [84] | Substitution of Ser for Cys-163 (catalytic triad) yielded an enzyme that retained activity on some substrates, while a substitution of Gly at this position resulted in a completely inactive enzyme |
| TEV protease | PA | C04 | CHD | Nε | C151S (eq. C339S) | ---<br>+ | [85]<br><br>[86] | Ser can replace Cys at position 339, although proteolytic activity is greatly reduced.<br><br>Mutation of the tobacco etch virus (TEV) protease nucleophile from cysteine to serine causes an approximately ~10^4-fold loss in activity. Ten rounds of directed evolution of the mutant, TEVSer, overcame the detrimental effects of nucleophile exchange to recover near-wild-type activity in the mutant TEVSerX. Rather than respecialising TEV to the new nucleophile, all the enzymes along the evolutionary trajectory also retained the ability to use the original cysteine nucleophile. |
| **esterases & other enzymes** | | | | | | | | |
| Dieckmann cyclase | | | CHD | Nε | C89S | --- | [21] | Cys to Ser replacement did not demonstrate the anticipated hydrolytic activity. |
| DLH dienelactone hydrolase | | | CHD | Nε | C123S | -- | [87] | C123S DLH (Km = 9900±2300 µM; Vmax = 4.4±0.8 µmol/min-mg) displays burst kinetics with p-nitrophenyl acetate and is 10% as active as DLH (Km = 170±7 µM; Vmax = 21.1±0.4 µmol/min-mg). C123A DLH is inactive. |
| BAAT bile acid conjugating enzyme | | | CHD | Nε | C235S | +/- | [88]<br><br><br><br>[89] | a change from 235Cys to 235Ser alters BAAT selectivity for glycine and taurine (Sfakianos et al. 2002). Wild-type human BAAT can utilize either taurine or glycine (Falany et al. 1994), but the 235Ser mutant, while retaining its BAAT activity with taurine, has a substantially reduced BAAT activity with glycine as the substrate. Moreover, this mutant also exhibits a marked hydrolase activity against bile acid-CoA substrate (Sfakianos et al. 2002)<br><br>Although substitution of Cys-235 with serine generated an hBAT mutant with lower N-acyltransferase activity, it substantially increased the bile acid-CoA thioesterase activity compared with wild type<br>From amino acid sequence alignment (Fig. 1), hBAT is significantly related to acyl-CoA thioesterases. |
| GrgF hydrolase | | | CHD | Nε | C115S | +/- | [20] | C115S mutant functions as a thioesterase, while losing the ability to perform chain fusion. Importantly, the hydrolysis occurs much slower in the wild- type enzyme-catalyzed reaction (Figure 4D, traces iv to vi), and no hydrolyzed product was detected from the C115A mutant |
| ObiF nonribosomal peptide synthetase | | | CHD | Nε | C1141S | +/- | [90] | The C1141S mutation converted ObiF from a ‘β-lactone synthase’ to a classic hydrolase that produced only Obi-COOH from the in vitro reaction of the holo-ObiF C1141S mutant and<br>holo-ObiD with β-OH-p-NO2-homoPhe and 2,3-DHB (Fig. 3b,c; Supplementary Fig. 15; Supplementary Table 5). The C1141A mutation brought the catalytic activity of ObiF to a near halt, with only trace amounts of Obi-COOH detected by LC–MS |

## Table S5. Evolutionary cases of Ser/Cys nucleophile switch.

| Enzyme | MEROPS clan | MEROPS fam. | Triad | Triad type | Natural variant | Activity loss/ gain | Ref. | Catalytic effect |
|---|---|---|---|---|---|---|---|---|
| **General** | | | | | | | | |
| chymotrypsin proteases and 3C viral proteases | PA | | | Nε | | + | [91]<br><br>[92] | Unexpectedly, the 2a and 3c subclasses of viral cysteine proteases are, respectively, homologous to the small and large structural subclasses of trypsin-like serine proteases.<br><br>Evidence is presented, based on sequence comparison and secondary structure prediction, of structural and evolutionary relationship between chymotrypsin-like serine proteases, cysteine proteases of positive strand RNA viruses (3C proteases of picornaviruses and related enzymes of como-, nepo- and potyviruses) and putative serine protease of a sobemovirus. |
| thioesterases | | | | Nε | | | [93] | Some TEs deviate from the canonical Ser-His-Asp triad (see alignment in ESI\u2020) and contain a Cys in place of the Ser. For example, the TE domain from pyochelin biosynthesis in Pseudomonas aeruginosa50 and Streptomyces venezuelae51 both contain a GXCXG motif rather than the canonical GXSXG motif. Mutation of the native Ser nucleophile to Cys in both myristoyl- ACP TE from Vibrio harveyi52 and rat mammary gland TE II53 generates catalytically active TEs, suggesting that native Cys containing TEs are likely active. Deacylation of the active site Cys in Ser to Cys mutants has been shown in vitro to be rate limiting.52,53 The Ser to Cys and His to Arg double mutant has even slower rates of deacylation and has been used to trap the acyl-TE intermediate for the TE from the tautomycin biosynthetic pathway.28 |
| metacaspases | CD | | | - | CH->SK | | [94]<br><br>[95] | In several metacaspases from clan CD the catalytic cysteine has been substituted with a serine or threonine (e.g. T. brucei homologs of metacaspase 2), but the prediction of these nucleophiles is complicated by the divergence of these proteins from a solved structure.<br><br>The bioinformatics analysis showed that the peculiar feature of PfMCA3 was the replacement of the catalytic cysteine and histidine residues by serine (S) and lysine (K) at the consensus position, respectively. The plausible explanation for such replacement could be that the serine1865 acts as catalytic residue in the active site in place of cysteine and might participate in peptidase activity. |
| ECP1/SERA | CA | C01 | C/SHN | Nδ | | | [94] | Note that humans have two C1 peptidases out of a total of 13 in which serines replace the cysteine nucleophile; these proteins appear to be involved in some cases of renal failure [58]. Family C1 classes containing these unusual proteins include the ECP1/SERA proteins from Plasmodium, that are key to host cell egress through an unknown mechanism [59], and isoforms of the T. congolense cathepsin-B-like peptidases and Theileria annulata cathepsin-L-like enzymes [2]. |
| **Specific cases** | | | | | | | | |
| congopain | CA | C01 | C/SHN | Nδ | C25S | -/+ | [96] | Surprisingly, a variant with the catalytic triad Ser25, His159 and Asn175 was shown to be active against classical cysteine peptidase substrates and inhibited by E-64, a class-specific cysteine protease inhibitor.<br>one of the most often recurring substitutions of the congopain-like genes is that of the active site cysteine (Cys25) for a serine (Ser25). Also, the active site His191 (congopain numbering, Fig. 1) was less often substituted by a Ser or Tyr. According to the methods used to estimate the prevalence of the genes bearing these substitutions, it was found that 40–60% of the congopain-like genes present this type of mutation [23].<br>Active site titration was conducted in order to determine the concentration of active enzyme in each recombinantly expressed CP variant preparation using E-64 as an active site titrant. For C2 and CPSYN, only 20% of the respective proteins showed enzymatic activity. CPSHN, however, contained 80% active enzyme. Surprisingly, CPSHN and CPSYN with active site Cys to Ser mutations showed activity against Z-Phe-Arg-AMC and inhibition by E-64. |
| SERA5 | CA | C01 | C/SHN | Nδ | C596S | ---<br>? | [97]<br><br>[98] | All SERA proteins possess a central domain that has homology to papain except in the case of SERA5 (and some other SERAs), where the active site cysteine has been replaced with a serine. To investigate if this domain retains catalytic activity, we expressed, purified, and refolded a recombinant form of the SERA5 enzyme domain. This protein possessed chymotrypsin-like proteolytic activity as it processed substrates downstream of aromatic residues, and its activity was reversed by the serine protease inhibitor 3,4-diisocoumarin.<br>In *P. falciparum*, six of the nine SERAs have an active site serine (SERA1–5 and SERA9), whereas the other three have an active site cysteine.<br><br>In the present study, we provide evidence for the enzymatic activity of PfSERA5 ~ 50 kDa protein fragment (PfSERA5P50) and its inhibition by two |

| | | | | | | | | | |
|---|---|---|---|---|---|---|---|---|---|
| | | | | | | | | | peptides derived from the 6 kDa fragment.<br>The recombinant PfSERA5P 50 was effectively able to hydrolyze the substrate in a dose dependent manner with a Vmax of 250 pmol/min and Km of 13.75 uM.<br>The recombinant protein lost its activity upon heat denaturation. To rule out any contamination by the bacterial proteases during purification catalytic serine residue was replaced by an alanine using site directed mutagenesis. Mutated protein did not show any protease activity |
| | | | | | | | | [99] | whilst rSERA5wt and rSERA5-A were consistently purified as predominantly full-length proteins, the rSERA5-C variant expressed and purified under identical conditions was reproducibly obtained in a partially degraded form<br>substitution of the SERA5 Ser596 residue in a manner predicted to ablate any catalytic activity did not result in detectable compensatory changes in expression of *SERA5* or other members of the *SERA* gene family, consistent with the notion that SERA5 function is not affected by the Ser596Ala substitution.<br>we were unable to detect hydrolytic activity associated with the parasite-derived protein or with recombinant SERA5 containing the wild-type Ser565 residue, either in intact form or following digestion *in vitro* with rPfSUB1. These results contrast with those of Hodder *et al*. (2003) and Kanodia *et al*. (2014) who demonstrated a chymotrypsin-like protease activity associated with a refolded, *E. coli*-derived recombinant form of the SERA5 papain-like domain. The reason for this discrepancy is unclear. However, we found that a recombinant protein containing a Cys substitution of Ser596 (rSERA5-C) displayed robust peptidase activity with the characteristics of a papain-like cysteine protease, including activation by DTT and sensitivity to E64. |
| | | | | | | | | [100] | The possibility of protease activity for Group IV SERA was dismissed by reverse genetics analysis of Pf-SERA5; as substituting the serine residue for alanine had no phenotypic consequence [22]. |
| EnCL | CA | C01 | C/SHN | Nδ (pred) | C25S | + | | [101] | All but one of the *EnCLs* characterised in this study possess a typical catalytic triad of cysteine peptidase active site (Cys/His/Asn); the exception is *EnCL2*, in which the active site cysteine (Cys25) is replaced by serine (Ser25). However, as described for congopain (cathepsin L-like peptidase) in *Trypanosoma congolense*, Cys-to-Ser mutation need not necessarily affect the proteolytic ability of an enzyme [53]. It has been also suggested that Cys-to-Ser mutation in the cysteine peptidases of parasites might be a way to moderate oxidation of the active site in case parasites have to deal with an oxygen-rich environment, such as blood or external environment [17]. |
| testin | CA | C01 | C/SHN | Nδ (pred) | C25S | --- | | [102] | the active-site cysteine residue has been changed to a serine. While proteolytic activity is still theoretically possible, no such activity has been detected |
| silicatein | CA | C01 | SHN | Nδ | S26 | + | | [103] | Interestingly, the catalytic cysteine (sulfhydryl) residue at the active site of the proteases is replaced in silicatein α with a serine (hydroxyl). Consistent with this replacement, we have found that the silicateins do not display esterase activity when tested with synthetic chromogenic substrates. |
| | | | | | | | | [104] | Recent site-directed mutagenesis results (22) confirm the requirement for the specific Ser26 and His165 residues of silicatein α for catalysis of the siloxane polymerization described here. |
| | | | | | | | | [105] | Replacement of either of two specific side chain functionalities (Ser-26 and His-165) significantly diminishes catalysis, supporting a reaction mechanism analogous to that of a well-known enzyme that is highly homologous to the silica protein.<br>The catalytic activity of the mutant proteins is reduced by an order of magnitude (Figure 1), to a residual value somewhat higher than that of the heat-denatured protein. |
| | | | | | | | | [106] | The silicateins are distinguished from the cathepsins by the replacement of the first amino acid residue in the catalytic triad, cysteine by serine<br>silicatein not only mediates polymerization of silicate, but also displays proteolytic activity which is specific for cathepsin L enzymes, thus underscoring the high relationship of the silicateins to cathepsin L. |
| Otu deubiquitinase | CA | C85 | C/SHD | Nδ (pred) | C40S | --- | | [107] | We have discovered that the two Drosophila melanogaster homologues of human OTUD4, CG3251 and Otu, contain a serine instead of a cysteine in the catalytic OTU (ovarian tumor) domain. DUBs that are serine proteases instead of cysteine- or metallo-proteases have not been described. In line with this, neither CG3251 nor Otu protein were active to cleave ubiquitin chains. Re-introduction of a cysteine in the catalytic center did not render the enzymes active, indicating that further critical features for ubiquitin binding or cleavage have been lost in these proteins. Sequence analysis of OTUD4 homologues from various other species showed that within this OTU subfamily, loss of the catalytic cysteine has occurred frequently in presumably independent events. |

## Table S6. Correspondence between ESTHER and other databases.

| ESTHER[6] | PFAM[11] | Arpigny & Jaeger[108,109] | CAZY[7] | MEROPS[5] | ThYme[8] | LED[9] | Other |
|---|---|---|---|---|---|---|---|
| 5_AlphaBeta_hydrolase | PF02230 PF00326 | | | | | | |
| 6_AlphaBeta_hydrolase | PF00561 PF12697 PF12146 | | | | | | |
| A85-EsteraseD-FGH | PF00756 | | | | TE22 | | |
| A85-Feruloyl-Esterase | PF00756 | | | | | abH32.01 abH32.02 | |
| A85-IroE-IroD-Fes-Yiel | PF00756 | | | | | | |
| A85-Mycolyl-transferase | PF00756 | | CE1 | | | abH33.01 abH33.02 abH33.03 | |
| ABHD6-Lip | PF00561 PF12697 | Family_V.1 | | | | | |
| ABHD10 | PF00561 | | | | | | |
| ABHD11-Acetyl_transferase | PF00561 PF12697 | | | | | abH07.01 | |
| Abhydrolase_7 | PF12715 | | | | | | |
| abh_upf00227 | PF05728 | | | | | | |
| Acetyl-esterase_deacetylase | PF05448 | | CE7 | | | abH26.01 | |
| Acetylxylan_esterase | PF01083 | | CE5 | | | | |
| AChE | PF00135 | | | | | abH01.04 | |
| Acidic_Lipase | PF00561 | | | | | abH14.02 | |
| Aclacinomycin-methylesterase_RdmC | PF00561 | | | | | | |
| ACPH_Peptidase_S9 | PF00326 | | | S09 | | | |
| Acyl-CoA_Thioesterase | PF08840 | | | | TE2 | | |
| AHL-acylase | PF12697 | | | | | | |
| AlphaBeta_hydrolase | PF00561 PF12146 PF05728 | | | | | | |
| Atu1826-like | PF20408 PF02129 | | | | | | |
| Bacterial_Est97 Family_XVI | | | | | | | |
| Bacterial_esterase | PF12697 | | | | | abH13.01 | |
| Bacterial_lip_FamI.1 | PF00561 | Family I.1 | | | | abH15.02 | |
| Bacterial_lip_FamI.2 | PF00561 | Family_I.2 | | | | abH15.02 | |
| Bacterial_lip_FamI.3 | PF00353 | Family_I.3 | | | | abH24.01 | |
| Bacterial_lip_FamI.5 | | Family_I.5 | | | | abH15.01 | |
| Bacterial_lip_FamI.6 | | Family_I.6 | | | | abH15.01 | |
| BCHE | PF00135 | | | | | abH01.04 | |
| BioH | PF00561 | | | | | | |
| BlEst2-lipase-like | | | | | | | |

| | | | | | | | |
|---|---|---|---|---|---|---|---|
| Canar_LipB | | | | | | abH37.01 | |
| CarbLipBact_1 | PF12146 | Family_XIII | | | | abH11.01 | |
| CarbLipBact_2 | PF12146 | Family_XIII | | | | | |
| Carbon-carbon_bond_hydrolase | PF00561 PF12697 | | | S33 | | abH08.08 abH08.11 | |
| Carboxymethylbutenolide_lactonase | PF00561 PF12146 | Family_V.2 | | | | | |
| Carboxypeptidase_S10 | PF00450 | | | S10 | | abH34.01 abH34.02 | |
| Carb_B_Arthropoda | PF00135 | | | | | | |
| Carb_B_Bacteria | PF00135 | Family_VII | | | | abH01.05 | |
| Carb_B_Chordata | PF00135 | | | | | abH01.02 | |
| CFTR-inhibitory-factor_Cif | PF12697 | | | | | | |
| Cholesterol_esterase | PF00135 | | | | | abH01.03 | |
| CIB-CCG1-interacting-factor-B | PF00561 PF12697 | | | | | abH08.14 | |
| Cocaine_esterase | PF02129 | | | | | abH30.01 | |
| Cutinase | PF01083 | | CE5 | | | abH36.03 | |
| DLH-S | PF01738 | | | | | | |
| DPP4N_Peptidase_S9 | PF00326 | | | S09 | | abH27.01 | |
| Duf_452 | PF04301 | | | | | | |
| Duf_915 | PF06028 | | | | | | |
| Duf_1100-S | PF06500 PF12146 | | | | | | |
| Duf_1749 | PF08538 | | | | | | |
| Duf_2235 | PF09994 | | | | | | |
| Est-OsmC | PF12146 | | | | | | |
| Esterase_phb | PF10503 | | CE1 | | | | |
| Esterase_phb_PHAZ | PF10503 | | | | | | |
| FSH1 | PF03959 | | | | | | |
| Fungal-Bact_LIP | PF03583 | Family_X.2 | | | | abH38.01 | |
| Fungal_carboxylesterase_lipase | PF00135 | | | | | abH03.01 | |
| Glucuronoyl_esterase | | | CE15 | | | | |
| GTSAGmotif | PF07859 | | | | | | |
| Haloperoxidase | PF00561 PF12697 PF00598 | | | | | abH08.09 | |
| HNLyase_Bact | PF12697 | | | | | | |
| HOD-cofactorfree-dioxygenase | PF00561 PF12697 | | | | | | |
| Homoserine_transacetylase | PF00561 | | | | | | |
| Homoserine_transacetylase_like_est | PF00561 | | | | | | |
| Hormone-sensitive_lipase_like | PF07859 PF00326 PF20434 | Family_IV | | S09 | | abH04.01 abH06.01 | |
| Hydrolase_RBBP9_YdeN | PF06821 | | | | | | |

| | | | | | | | |
|---|---|---|---|---|---|---|---|
| Hydroxynitrile_lyase | PF00561 PF12697 | | | | | abH12.01 | |
| Insect_Phospholipase | PF00151 | | | | | | |
| Juvenile_hormone_esterase | PF00135 | | | | | abH01.07 | |
| Kynurenine-formamidase | PF07859 | | | | | | |
| Lactobacillus_peptidase | PF02129 | | | S15 | | | |
| Lipase_2 | PF01674 | Family_I.7 | | | | abH18.01 | |
| Lipase_3 | PF01764 | Family_XI | | | | abH23.01 | |
| Lipoprotein_Lipase | PF00151 | | | | | | |
| lpg2422 | PF18532 | | | | | | |
| Lysophospholipase_carboxylesterase | PF02230 PF01738 | Family_VI | | | TE21 | abH22.01 abH22.03 | |
| MenH_SHCHC | PF12697 | | | | | | |
| Mg-chelatase_BchO | PF12697 | | | | | | |
| Monoglyceridelipase_lysophospholip | PF12146 | | | | | | |
| MpaH | PF12697 | | | | | | |
| PAF-Acetylhydrolase | PF03403 | | | | | | |
| Palmitoyl-protein_thioesterase | PF02089 | | | | TE20 | abH19.01 abH19.02 | |
| Pancreatic_lipase | PF00151 | | | | | abH20.03 | |
| PC-sterol_acyltransferase | PF02450 | Family_XIV | | | | | |
| Pectinacetylesterase-Notum | PF03283 | | | | | | |
| Pectin_methylesterase | | | CE19 | | | | |
| PGAP1 | | | | | | | |
| PHAZ7_phb_depolymerase | PF01674 | Family_IX | | | | | |
| Plant_carboxylesterase | PF07859 | | | | | abH04.04 | |
| Plant_lipase_EDS1-like | PF01764 | | | | | | |
| Plant_phospholipase | PF01764 | | | | | | |
| PMH_Peptidase_S9 | PF00326 | | | | | | |
| PolyAspartate-hydrolase | | | | | | | |
| Polyesterase-lipase-cutinase | PF12695 PF12740 PF01738 PF03403 | Family_III | | | | abH25.01 | |
| Proline_iminopeptidase | PF00561 PF12146 | | | S33 | | abH08.13 abH09.03 | |
| Prolylcarboxypeptidase | PF05577 | | | S28 | | | |
| Prolyl_oligopeptidase_S9 | PF00326 | | | | | | |
| RsbQ-like | PF00561 PF12697 | | | | | abH08.13 | |
| S9N_PPCE_Peptidase_S9 | PF00326 | | | S09 | | abH28.01 | |
| S9N_PREPL_Peptidase_S9 | PF00326 | | | | | | |
| Tannase | PF07519 | | | | | | |
| Tannase_Bact | PF20434 | | | | | | |

| | | | | | | | |
|---|---|---|---|---|---|---|---|
| Thioesterase | PF00975 | | | | TE16 TE17 TE18 | abH07.01 | |
| Thioesterase_acyl-transferase | PF02273 | | | | TE19 | abH35.01 | |
| Thiohydrolase | PF12146 | | | | | | |
| Valacyclovir-hydrolase | PF00561 | | | | | | |
| Zearalenone-hydrolase | PF00561 | | | | | | |
| HE | PF03996 | | | | | | |
| AOAH | PF00657 | | | | | | SGNH |
| HE | PF03996 | | | | | | |
| HE | PF03996 | | | | | | |
| EstA | PF00657 | | | | | | SGNH |
| PAFAH1B2 | PF13472 | | | | | | SGNH |
| HE | PF03996 | | | | | | |
| CheB | PF01339 | | | | | | |
| HE | PF03996 | | | | | | |
| AXE2C | PF13472 | | CE2 | | | | SGNH |
| PLA1 | PF02253 | | | | | | |
| PAFAH1B3 | PF13472 | | | | | | SGNH |
| rha1 | PF13472 PF00657 | | CE12 | | | | SGNH |
| SMc01033_Esterase | PF13472 | | | | | | SGNH |
| GDSL_lipolytic_enzyme | PF13472 | | | | | | SGNH |
| GDSL_lipolytic_enzyme | PF13472 | | CE3 | | | | SGNH |
| TesA | PF13472 | | | | TE3 | | SGNH |
| CheB | PF01339 | | | | | | |
| TesA | PF00657 | | | | TE3 | | SGNH |
| At4g34215 | PF03629 | | CE6 | | | | SGNH (manually added) |

**Table S7. Distribution of third triad residue type in peptidases and esterases / α/β-hydrolases.**

**(A) unweighted cases**

| | Nε-triads | | | | Nδ-triads | | | | |
|---|---|---|---|---|---|---|---|---|---|
| | **Asp** | **Glu** | **Asn** | **Gln** | **Asp** | **Glu** | **Asn** | **Gln** | **Total** |
| **serine peptidases** | 3152 | 28 | 7 | 0 | 0 | 0 | 0 | 0 | **3187** |
| **cysteine peptidases** | 14 | 130 | 0 | 0 | 74 | 27 | 348 | 0 | **593** |
| **serine esterases & α/β-hydrolases** | 2578 | 774 | 4 | 0 | 9 | 0 | 0 | 0 | **3365** |
| **cysteine esterases & α/β-hydrolases** | 14 | 0 | 0 | 0 | 0 | 0 | 0 | 0 | **14** |
| **PFAM annotated serine esterases & α/β-hydrolases** | 2375 | 736 | 4 | 0 | 9 | 0 | 0 | 0 | **3124** |
| **PFAM annotated cysteine esterases & α/β-hydrolases** | 7 | 0 | 0 | 0 | 0 | 0 | 0 | 0 | **7** |

**(B) weighted cases**

| | Nε-triads | | | | Nδ-triads | | | | |
|---|---|---|---|---|---|---|---|---|---|
| | **Asp** | **Glu** | **Asn** | **Gln** | **Asp** | **Glu** | **Asn** | **Gln** | **Total** |
| **serine peptidases** | 130 | 7 | 1 | 0 | 0 | 0 | 0 | 0 | **138** |
| **cysteine peptidases** | 5 | 16 | 0 | 0 | 20.5 | 3.5 | 26 | 0 | **71** |
| **serine esterases & α/β-hydrolases** | 390 | 57 | 1 | 0 | 2 | 0 | 0 | 0 | **450** |
| **cysteine esterases & α/β-hydrolases** | 8 | 0 | 0 | 0 | 0 | 0 | 0 | 0 | **8** |
| **PFAM annotated serine esterases & α/β-hydrolases** | 343 | 47 | 1 | 0 | 2 | 0 | 0 | 0 | **393** |
| **PFAM annotated cysteine esterases & α/β-hydrolases** | 5 | 0 | 0 | 0 | 0 | 0 | 0 | 0 | **5** |

## References


[1] K. Kawamura, T. Yamada, K. Kurihara, T. Tamada, R. Kuroki, I. Tanaka, H. Takahashi, N. Niimura, *Acta Crystallogr D Biol Crystallogr* **2011**, *67*, 140–148.

[2] P. A. Wood, F. H. Allen, E. Pidcock, *CrystEngComm* **2009**, *11*, 1563–1571.

[3] H. M. Berman, J. Westbrook, Z. Feng, G. Gilliland, T. N. Bhat, H. Weissig, I. N. Shindyalov, P. E. Bourne, *Nucleic Acids Res* **2000**, *28*, 235–242.

[4] W. Li, A. Godzik, *Bioinformatics* **2006**, *22*, 1658–1659.

[5] N. D. Rawlings, A. J. Barrett, R. Finn, *Nucleic Acids Res* **2016**, *44*, D343-350.

[6] N. Lenfant, T. Hotelier, E. Velluet, Y. Bourne, P. Marchot, A. Chatonnet, *Nucleic Acids Res* **2013**, *41*, D423-429.

[7] E. Drula, M.-L. Garron, S. Dogan, V. Lombard, B. Henrissat, N. Terrapon, *Nucleic Acids Research* **2022**, *50*, D571–D577.

[8] D. C. Cantu, Y. Chen, M. L. Lemons, P. J. Reilly, *Nucleic Acids Research* **2011**, *39*, D342–D346.

[9] M. Fischer, *Nucleic Acids Research* **2003**, *31*, 319–321.

[10] The UniProt Consortium, A. Bateman, M.-J. Martin, S. Orchard, M. Magrane, R. Agivetova, S. Ahmad, E. Alpi, E. H. Bowler-Barnett, R. Britto, B. Bursteinas, H. Bye-A-Jee, R. Coetzee, A. Cukura, A. Da Silva, P. Denny, T. Dogan, T. Ebenezer, J. Fan, L. G. Castro, P. Garmiri, G. Georghiou, L. Gonzales, E. Hatton-Ellis, A. Hussein, A. Ignatchenko, G. Insana, R. Ishtiaq, P. Jokinen, V. Joshi, D. Jyothi, A. Lock, R. Lopez, A. Luciani, J. Luo, Y. Lussi, A. MacDougall, F. Madeira, M. Mahmoudy, M. Menchi, A. Mishra, K. Moulang, A. Nightingale, C. S. Oliveira, S. Pundir, G. Qi, S. Raj, D. Rice, M. R. Lopez, R. Saidi, J. Sampson, T. Sawford, E. Speretta, E. Turner, N. Tyagi, P. Vasudev, V. Volynkin, K. Warner, X. Watkins, R. Zaru, H. Zellner, A. Bridge, S. Poux, N. Redaschi, L. Aimo, G. Argoud-Puy, A. Auchincloss, K. Axelsen, P. Bansal, D. Baratin, M.-C. Blatter, J. Bolleman, E. Boutet, L. Breuza, C. Casals-Casas, E. de Castro, K. C. Echioukh, E. Coudert, B. Cuche, M. Doche, D. Dornevil, A. Estreicher, M. L. Famiglietti, M. Feuermann, E. Gasteiger, S. Gehant, V. Gerritsen, A. Gos, N. Gruaz-Gumowski, U. Hinz, C. Hulo, N. Hyka-Nouspikel, F. Jungo, G. Keller, A. Kerhornou, V. Lara, P. Le Mercier, D. Lieberherr, T. Lombardot, X. Martin, P. Masson, A. Morgat, T. B. Neto, S. Paesano, I. Pedruzzi, S. Pilbout, L. Pourcel, M. Pozzato, M. Pruess, C. Rivoire, C. Sigrist, K. Sonesson, A. Stutz, S. Sundaram, M. Tognolli, L. Verbregue, C. H. Wu, C. N. Arighi, L. Arminski, C. Chen, Y. Chen, J. S. Garavelli, H. Huang, K. Laiho, P. McGarvey, D. A. Natale, K. Ross, C. R. Vinayaka, Q. Wang, Y. Wang, L.-S. Yeh, J. Zhang, P. Ruch, D. Teodoro, *Nucleic Acids Research* **2021**, *49*, D480–D489.

[11] J. Mistry, S. Chuguransky, L. Williams, M. Qureshi, G. A. Salazar, E. L. L. Sonnhammer, S. C. E. Tosatto, L. Paladin, S. Raj, L. J. Richardson, R. D. Finn, A. Bateman, *Nucleic Acids Research* **2021**, *49*, D412–D419.

[12] E. Zeiler, A. List, F. Alte, M. Gersch, R. Wachtel, M. Poreba, M. Drag, M. Groll, S. A. Sieber, *PNAS* **2013**, *110*, 11302–11307.

[13] A. R. Wagh, K. Bose, *Biosci Rep* **2018**, *38*, BSR20181072.

[14] K. Sugawara, N. N. Suzuki, Y. Fujioka, N. Mizushima, Y. Ohsumi, F. Inagaki, *Journal of Biological Chemistry* **2005**, *280*, 40058–40065.

[15] S. Ye, B. Loll, A. A. Berger, U. Mülow, C. Alings, M. C. Wahl, B. Koksch, *Chem Sci* **2015**, *6*, 5246–5254.

[16] D. A. Matthews, P. S. Dragovich, S. E. Webber, S. A. Fuhrman, A. K. Patick, L. S. Zalman, T. F. Hendrickson, R. A. Love, T. J. Prins, J. T. Marakovits, R. Zhou, J. Tikhe, C. E. Ford, J. W. Meador, R. A. Ferre, E. L. Brown, S. L. Binford, M. A. Brothers, D. M. DeLisle, S. T. Worland, *Proc Natl Acad Sci U S A* **1999**, *96*, 11000–11007.
[17] V. Fülöp, Z. Böcskei, L. Polgár, *Cell* **1998**, *94*, 161–170.
[18] D. Pathak, D. Ollis, *Journal of Molecular Biology* **1990**, *214*, 497–525.
[19] M. F. Chek, S.-Y. Kim, T. Mori, H. Arsad, M. R. Samian, K. Sudesh, T. Hakoshima, *Sci Rep* **2017**, *7*, 5312.
[20] W.-G. Wang, H. Wang, L.-Q. Du, M. Li, L. Chen, J. Yu, G.-G. Cheng, M.-T. Zhan, Q.-F. Hu, L. Zhang, M. Yao, Y. Matsuda, *J Am Chem Soc* **2020**, *142*, 8464–8472.
[21] D. P. Cogan, J. Ly, S. K. Nair, *ACS Chem. Biol.* **2020**, *15*, 2783–2791.
[22] E. J. Drake, B. R. Miller, C. Shi, J. T. Tarrasch, J. A. Sundlov, C. Leigh Allen, G. Skiniotis, C. C. Aldrich, A. M. Gulick, *Nature* **2016**, *529*, 235–238.
[23] P. Emsley, K. Cowtan, *Acta Crystallogr D Biol Crystallogr* **2004**, *60*, 2126–2132.
[24] F. M. Richards, *Journal of Molecular Biology* **1974**, *82*, 1–14.
[25] M. D. Winn, C. C. Ballard, K. D. Cowtan, E. J. Dodson, P. Emsley, P. R. Evans, R. M. Keegan, E. B. Krissinel, A. G. W. Leslie, A. McCoy, S. J. McNicholas, G. N. Murshudov, N. S. Pannu, E. A. Potterton, H. R. Powell, R. J. Read, A. Vagin, K. S. Wilson, *Acta Crystallogr D Biol Crystallogr* **2011**, *67*, 235–242.
[26] I. W. Davis, A. Leaver-Fay, V. B. Chen, J. N. Block, G. J. Kapral, X. Wang, L. W. Murray, W. B. Arendall, J. Snoeyink, J. S. Richardson, D. C. Richardson, *Nucleic Acids Res* **2007**, *35*, W375-383.
[27] R. P. Joosten, J. Salzemann, V. Bloch, H. Stockinger, A.-C. Berglund, C. Blanchet, E. Bongcam-Rudloff, C. Combet, A. L. Da Costa, G. Deleage, M. Diarena, R. Fabbretti, G. Fettahi, V. Flegel, A. Gisel, V. Kasam, T. Kervinen, E. Korpelainen, K. Mattila, M. Pagni, M. Reichstadt, V. Breton, I. J. Tickle, G. Vriend, *J Appl Crystallogr* **2009**, *42*, 376–384.
[28] A. J. M. Ribeiro, G. L. Holliday, N. Furnham, J. D. Tyzack, K. Ferris, J. M. Thornton, *Nucleic Acids Res* **2018**, *46*, D618–D623.
[29] N. Nagano, N. Nakayama, K. Ikeda, M. Fukuie, K. Yokota, T. Doi, T. Kato, K. Tomii, *Nucleic Acids Res* **2015**, *43*, D453–D458.
[30] J. N. Higaki, L. B. Evnin, C. S. Craik, *Biochemistry* **1989**, *28*, 9256–9263.
[31] M. E. Wilke, J. N. Higaki, C. S. Craik, R. J. Fletterick, *Journal of Molecular Biology* **1991**, *219*, 511–523.
[32] H. Yokosawa, S. Ojima, S. Ishii, *The Journal of Biochemistry* **1977**, *82*, 869–876.
[33] Y. Hanakawa, N. M. Schechter, C. Lin, L. Garza, H. Li, T. Yamaguchi, Y. Fudaba, K. Nishifuji, M. Sugai, M. Amagai, J. R. Stanley, *J. Clin. Invest.* **2002**, *110*, 53–60.
[34] C. S. Hahn, J. H. Strauss, *J Virol* **1990**, *64*, 3069–3073.
[35] T. J. Chambers, R. C. Weir, A. Grakoui, D. W. McCourt, J. F. Bazan, R. J. Fletterick, C. M. Rice, *Proc. Natl. Acad. Sci. U.S.A.* **1990**, *87*, 8898–8902.
[36] K. E. Neet, D. E. Koshland, *Proc. Natl. Acad. Sci. U.S.A.* **1966**, *56*, 1606–1611.
[37] J. Arima, S. Tokai, M. Chiba, T. Ichiyanagi, Y. Yabuta, N. Mori, T. Aimi, *Bioscience, Biotechnology, and Biochemistry* **2014**, *78*, 1856–1863.

[38] H. Usuki, Y. Uesugi, J. Arima, Y. Yamamoto, M. Iwabuchi, T. Hatanaka, *Chem. Commun.* **2010**, *46*, 580–582.
[39] R. A. L. Lassy, C. G. Miller, *J Bacteriol* **2000**, *182*, 2536–2543.
[40] G. Gibney, S. Camp, M. Dionne, K. MacPhee-Quigley, P. Taylor, *Proc. Natl. Acad. Sci. U.S.A.* **1990**, *87*, 7546–7550.
[41] A. Shafferman, C. Kronman, Y. Flashner, M. Leitner, H. Grosfeld, A. Ordentlich, Y. Gozes, S. Cohen, N. Ariel, D. Barak, *J Biol Chem* **1992**, *267*, 17640–17648.
[42] J. M. Turner, N. A. Larsen, A. Basran, C. F. Barbas, N. C. Bruce, I. A. Wilson, R. A. Lerner, *Biochemistry* **2002**, *41*, 12297–12307.
[43] J. Li, R. Szittner, Z. S. Derewenda, E. A. Meighen, *Biochemistry* **1996**, *35*, 9967–9973.
[44] M. Pazirandeh, S. S. Chirala, S. J. Wakil, *J Biol Chem* **1991**, *266*, 20946–20952.
[45] M. H. Tai, S. S. Chirala, S. J. Wakil, *Proc. Natl. Acad. Sci. U.S.A.* **1993**, *90*, 1852–1856.
[46] A. Witkowski, J. Naggert, H. E. Witkowska, Z. I. Randhawa, S. Smith, *J Biol Chem* **1992**, *267*, 18488–18492.
[47] A. A. Koch, D. A. Hansen, V. V. Shende, L. R. Furan, K. N. Houk, G. Jiménez-Osés, D. H. Sherman, *J Am Chem Soc* **2017**, *139*, 13456–13465.
[48] K. Huhtinen, J. O'Byrne, P. J. G. Lindquist, J. A. Contreras, S. E. H. Alexson, *Journal of Biological Chemistry* **2002**, *277*, 3424–3432.
[49] C. Holm, R. C. Davis, T. Østerlund, M. C. Schotz, G. Fredrikson, *FEBS Letters* **1994**, *344*, 234–238.
[50] M. L. Tjeenk, Y. B. M. Bulsink, A. J. Slotboom, H. M. Verheij, G. H. de Haas, G. Demleitner, F. Götz, *Protein Eng Des Sel* **1994**, *7*, 579–583.
[51] Y. Cen, W. Singh, M. Arkin, T. S. Moody, M. Huang, J. Zhou, Q. Wu, M. T. Reetz, *Nat Commun* **2019**, *10*, 3198.
[52] H. Wong, R. C. Davis, T. Thuren, J. W. Goers, J. Nikazy, M. Waite, M. C. Schotz, *J Biol Chem* **1994**, *269*, 10319–10323.
[53] G. P. Horsman, J. Ke, S. Dai, S. Y. K. Seah, J. T. Bolin, L. D. Eltis, *Biochemistry* **2006**, *45*, 11071–11086.
[54] R. G. P. M. Brok, I. U. Belandia, N. Dekker, J. Tommassen, H. M. Verheij, *Biochemistry* **1996**, *35*, 7787–7793.
[55] P. I. Clark, G. Lowe, *J. Chem. Soc., Chem. Commun.* **1977**, 923.
[56] P. I. Clark, G. Lowe, *Eur J Biochem* **1978**, *84*, 293–299.
[57] M. S. McQueney, B. Y. Amegadzie, K. D'Alessio, C. R. Hanning, M. M. McLaughlin, D. McNulty, S. A. Carr, C. Ijames, J. Kurdyla, C. S. Jones, *Journal of Biological Chemistry* **1997**, *272*, 13955–13960.
[58] A. D. Rowan, P. Mason, L. Mach, J. S. Mort, *J Biol Chem* **1992**, *267*, 15993–15999.
[59] R. Coulombe, P. Grochulski, J. Sivaraman, R. Ménard, J. S. Mort, M. Cygler, *EMBO J* **1996**, *15*, 5492–5503.
[60] J. P. Turkenburg, M. B. A. C. Lamers, A. M. Brzozowski, L. M. Wright, R. E. Hubbard, S. L. Sturt, D. H. Williams, *Acta Crystallogr D Biol Crystallogr* **2002**, *58*, 451–455.
[61] P. Y. T. Shibao, M. Ferro, F. F. P. de Paula, B. S. Lima, F. Henrique-Silva, *IJMS* **2021**, *22*, 11476.
[62] J. S. Arthur, S. Gauthier, J. S. Elce, *FEBS Lett* **1995**, *368*, 397–400.
[63] H. Masumoto, T. Yoshizawa, H. Sorimachi, T. Nishino, S. Ishiura, K. Suzuki, *Journal of Biochemistry* **1998**, *124*, 957–961.
[64] C. Wang, J. K. Barry, Z. Min, G. Tordsen, A. G. Rao, O.-A. Olsen, *Journal of Biological Chemistry* **2003**, *278*, 34467–34474.
[65] K.-H. Wu, P. C. Tai, *Journal of Biological Chemistry* **2004**, *279*, 901–909.

[66] L. A. Furgerson Ihnken, C. Chatterjee, W. A. van der Donk, *Biochemistry* **2008**, *47*, 7352–7363.
[67] O. K. Vatamaniuk, S. Mari, A. Lang, S. Chalasani, L. O. Demkiv, P. A. Rea, *Journal of Biological Chemistry* **2004**, *279*, 22449–22460.
[68] N. D. Romanyuk, D. J. Rigden, O. K. Vatamaniuk, A. Lang, R. E. Cahoon, J. M. Jez, P. A. Rea, *Plant Physiology* **2006**, *141*, 858–869.
[69] K.-L. Sheahan, C. L. Cordero, K. J. F. Satchell, *EMBO J* **2007**, *26*, 2552–2561.
[70] Y. Lee, B. S. Kim, S. Choi, E.-Y. Lee, S. Park, J. Hwang, Y. Kwon, J. Hyun, C. Lee, J. F. Kim, S. H. Eom, M. H. Kim, *Proc. Natl. Acad. Sci. U.S.A.* **2019**, *116*, 18031–18040.
[71] K. Kitadokoro, S. Kamitani, M. Miyazawa, M. Hanajima-Ozawa, A. Fukui, M. Miyake, Y. Horiguchi, *Proc. Natl. Acad. Sci. U.S.A.* **2007**, *104*, 5139–5144.
[72] Q. Yao, J. Cui, Y. Zhu, G. Wang, L. Hu, C. Long, R. Cao, X. Liu, N. Huang, S. Chen, L. Liu, F. Shao, *Proc. Natl. Acad. Sci. U.S.A.* **2009**, *106*, 3716–3721.
[73] L. A. Ivanoff, T. Towatari, J. Ray, B. D. Korant, S. R. Petteway, *Proc. Natl. Acad. Sci. U.S.A.* **1986**, *83*, 5392–5396.
[74] K. M. Kean, M. T. Howell, S. Grünert, M. Girard, R. J. Jackson, *Virology* **1993**, *194*, 360–364.
[75] T. Hämmerle, C. U. Hellen, E. Wimmer, *J Biol Chem* **1991**, *266*, 5412–5416.
[76] Z. Sárkány, L. Polgár, *Biochemistry* **2003**, *42*, 516–522.
[77] M. A. Lawson, B. L. Semler, *Proc. Natl. Acad. Sci. U.S.A.* **1991**, *88*, 9919–9923.
[78] K. C. Cheah, L. E. Leong, A. G. Porter, *J Biol Chem* **1990**, *265*, 7180–7187.
[79] J. A. Knott, D. C. Orr, D. S. Montgomery, C. A. Sullivan, A. Weston, *Eur J Biochem* **1989**, *182*, 547–555.
[80] R. Margis, L. Pinck, *Virology* **1992**, *190*, 884–888.
[81] K. Miyashita, M. Kusumi, R. Utsumi, S. Katayama, M. Noda, T. Komano, N. Satoh, *Protein Eng* **1993**, *6*, 189–193.
[82] R. Gosert, G. Dollenmaier, M. Weitz, *J Virol* **1997**, *71*, 3062–3068.
[83] B. A. Malcolm, S. M. Chin, D. A. Jewell, J. R. Stratton-Thomas, K. B. Thudium, R. Ralston, S. Rosenberg, *Biochemistry* **1992**, *31*, 3358–3363.
[84] M. J. Grubman, M. Zellner, G. Bablanian, P. W. Mason, M. E. Piccone, *Virology* **1995**, *213*, 581–589.
[85] W. G. Dougherty, T. D. Parks, S. M. Cary, J. F. Bazan, R. J. Fletterick, *Virology* **1989**, *172*, 302–310.
[86] T. Shafee, P. Gatti-Lafranconi, R. Minter, F. Hollfelder, *ChemBioChem* **2015**, *16*, 1866–1869.
[87] D. Pathak, G. Ashley, D. Ollis, *Proteins* **1991**, *9*, 267–279.
[88] B. M. Kirilenko, L. R. Hagey, S. Barnes, C. N. Falany, M. Hiller, *Genome Biol Evol* **2019**, *11*, 3256–3268.
[89] M. K. Sfakianos, L. Wilson, M. Sakalian, C. N. Falany, S. Barnes, *J Biol Chem* **2002**, *277*, 47270–47275.
[90] J. E. Schaffer, M. R. Reck, N. K. Prasad, T. A. Wencewicz, *Nat Chem Biol* **2017**, *13*, 737–744.
[91] J. F. Bazan, R. J. Fletterick, *Proc Natl Acad Sci U S A* **1988**, *85*, 7872–7876.
[92] A. E. Gorbalenya, A. P. Donchenko, V. M. Blinov, E. V. Koonin, *FEBS Lett* **1989**, *243*, 103–114.
[93] M. E. Horsman, T. P. A. Hari, C. N. Boddy, *Nat Prod Rep* **2016**, *33*, 183–202.

[94] H. J. Atkinson, P. C. Babbitt, M. Sajid, *Trends in Parasitology* **2009**, *25*, 573–581.
[95] B. Kumar, S. Verma, M. Kashif, R. Sharma, Atul, R. Dixit, A. P. Singh, V. Pande, A. K. Saxena, M. Abid, K. C. Pandey, *International Journal of Biological Macromolecules* **2019**, *138*, 309–320.
[96] D. Pillay, A. F. Boulangé, T. H. T. Coetzer, *Protein Expr Purif* **2010**, *74*, 264–271.
[97] A. N. Hodder, D. R. Drew, V. C. Epa, M. Delorenzi, R. Bourgon, S. K. Miller, R. L. Moritz, D. F. Frecklington, R. J. Simpson, T. P. Speed, R. N. Pike, B. S. Crabb, *J Biol Chem* **2003**, *278*, 48169–48177.
[98] S. Kanodia, G. Kumar, L. Rizzi, A. Pedretti, A. N. Hodder, S. Romeo, P. Malhotra, *Biochim Biophys Acta* **2014**, *1840*, 2765–2775.
[99] R. Stallmach, M. Kavishwar, C. Withers-Martinez, F. Hackett, C. R. Collins, S. A. Howell, S. Yeoh, E. Knuepfer, A. J. Atid, A. A. Holder, M. J. Blackman, *Mol Microbiol* **2015**, *96*, 368–387.
[100] N. Arisue, N. M. Q. Palacpac, T. Tougan, T. Horii, *Parasites Vectors* **2020**, *13*, 170.
[101] L. Jedličková, H. Dvořáková, J. Dvořák, M. Kašný, L. Ulrychová, J. Vorel, V. Žárský, L. Mikeš, *Parasit Vectors* **2018**, *11*, 142.
[102] D. P. Dickinson, *Crit Rev Oral Biol Med* **2002**, *13*, 238–275.
[103] K. Shimizu, J. Cha, G. D. Stucky, D. E. Morse, *Proc Natl Acad Sci U S A* **1998**, *95*, 6234–6238.
[104] J. N. Cha, K. Shimizu, Y. Zhou, S. C. Christiansen, B. F. Chmelka, G. D. Stucky, D. E. Morse, *Proceedings of the National Academy of Sciences* **1999**, *96*, 361–365.
[105] Y. Zhou, K. Shimizu, J. N. Cha, G. D. Stucky, D. E. Morse, *Angew Chem Int Ed Engl* **1999**, *38*, 779–782.
[106] W. E. G. Müller, A. Boreiko, X. Wang, S. I. Belikov, M. Wiens, V. A. Grebenjuk, U. Schlossmacher, H. C. Schröder, *Gene* **2007**, *395*, 62–71.
[107] M. Louis, K. Hofmann, M. Broemer, *PLoS One* **2015**, *10*, e0143227.
[108] J. L. Arpigny, K. E. Jaeger, *Biochem J* **1999**, *343 Pt 1*, 177–183.
[109] K.-E. Jaeger, T. Eggert, *Current Opinion in Biotechnology* **2002**, *13*, 390–397.
